# Dynamic evolution of EZHIP, an inhibitor of the Polycomb Repressive Complex 2 in mammals

**DOI:** 10.64898/2025.12.12.693809

**Published:** 2025-12-12

**Authors:** Pravrutha Raman, Hana Khan, Janet M. Young, Toshio Tsukiyama, Harmit S. Malik

**Affiliations:** 1 Division of Basic Sciences, Fred Hutchinson Cancer Center, Seattle, Washington 98109, USA;; 2 Department of Biochemistry, University of Washington, Seattle, Washington 98195, USA;; 3 Howard Hughes Medical Institute, Fred Hutchinson Cancer Center, Seattle, Washington 98109, USA

**Keywords:** histone methyl transferase inhibitor, histone mimic, gene duplication, pseudogenes, positive selection, gametogenesis, diversifying selection, Polycomb Repressive Complex (PRC2)

## Abstract

The Polycomb Repressive Complex 2 (PRC2) is an ancient, conserved chromatin-interacting complex that controls gene expression, facilitating differentiation and cellular identity during development. Its regulation is critical in most eukaryotes. EZHIP was recently characterized as a PRC2 inhibitor and ‘oncohistone mimic’ in mammals. Although *Ezhip* expression is typically restricted to the germline, its aberrant expression in pediatric brain tumors inhibits PRC2-mediated H3K27 methylation and drives disease progression. To gain a deeper understanding of its normal functions, we systematically examined *Ezhip* evolution across 70 mammals using comparative genomics, synteny analysis, and motif discovery. Bolstering previous work, we find that *Ezhip* originated and has been strictly retained on the X chromosome in placental mammals. In addition to the highly conserved H3K27M-like histone mimic motif, our motif analysis reveals seven previously unidentified EZHIP motifs, including a putative nuclear localization signal, and tandem repeats that are largely well-conserved in placental mammals, except in some lineages. We hypothesize that these motifs are also critical to EZHIP’s functions, including in PRC2 interaction and inhibition. We show that *Ezhip* has evolved under strong diversifying selection in primates and underwent dynamic expansions and losses across species. Some paralogs, such as *Ezhip2* in primates, also evolved under positive selection. Based on its evolutionary attributes and germ-cell expression, we propose that *Ezhip* arose and evolved rapidly due to inter-parental conflict over fetal development *in utero* in placental mammals. Our work provides a foundation for further investigations into EZHIP’s essential, potentially multifaceted roles in mammalian reproduction and disease.

## Introduction

The Polycomb Repressive Complex 2 (PRC2) is a highly conserved multiprotein complex that plays a central role in gene silencing and regulating gene expression during development and differentiation (Laugesen et al., 2019; Yu et al., 2019). The core subunits of PRC2 include EZH2 (enhancer of zeste homolog 2), the catalytic subunit responsible for methyltransferase activity, along with EED (embryonic ectoderm development), SUZ12 (suppressor of zeste 12), and RBBP4/7 (Retinoblastoma-binding Protein 4/7, also known as RbAp46/48) (Laugesen et al., 2019; Yu et al., 2019). Functionally, PRC2 is essential for establishing cellular memory and maintaining proper gene expression profiles during development by silencing genes involved in cell fate determination, including many developmental regulators and transcription factors (Yu et al., 2019). PRC2 catalyses the methylation of histone H3 at lysine 27 (H3K27me3) (Laugesen et al., 2019). This epigenetic mark is associated with the formation of facultative heterochromatin and transcriptional repression (Laugesen et al., 2019). PRC2 has also been shown to silence transposable elements in some species (Hisanaga et al., 2023; Shaver et al., 2010). In mammals, PRC2 plays critical roles in epigenetic processes, such as X chromosome inactivation, imprinting, and maintenance of stem cell identity (Yu et al., 2019). Dysregulation of PRC2, whether through loss or gain of function, has been linked to several diseases, including various cancers and developmental disorders, emphasizing the biological and clinical significance of this vital chromatin-modifying complex (Yu et al., 2019). Moreover, H3K27M ‘oncohistone’ mutations, which render H3 impervious to PRC2 activity, are associated with disease progression in several cancers (Lewis et al., 2013).

PRC2 activity is regulated by both internal feedback mechanisms and a variety of chromatin-associated factors, including post-translationally modified histones, noncoding RNAs, and accessory proteins, which help target PRC2 to specific locations or modify its catalytic activity (Kasinath et al., 2018; Yu et al., 2019). These include histone modifications such as H3K27me3 itself and H2AK119ub, as well as PRC2’s interactions with RNAs and nucleosome density, which control the timing and placement of gene silencing (Blackledge et al., 2014; Kasinath et al., 2018; Yu et al., 2019). A notable method of PRC2 regulation involves protein subunits that mimic unmodified histones to control the complex’s activity. For instance, PRC2 methylates accessory proteins JARID2 and PALI1 at specific lysine residues, whose structural resemblance to the H3K27me3 mark enables them to bind EED and allosterically activate PRC2’s enzymatic action (Kasinath et al., 2018). Other proteins, such as AEBP2, also interact with PRC2 via histone tail-like motifs, further refining chromatin association and gene repression (Kasinath et al., 2018).

Another ‘histone mimic’ that binds PRC2 is encoded by *Ezhip* (Enhancer of Zeste Homologs Inhibitory Protein; initially identified as *CXorf67*; also called *CATACOMB*, for catalytic antagonist of Polycomb). Instead of activating PRC2, EZHIP functions as a competitive inhibitor of the PRC2 complex by interfering with EZH2 activity (Hübner et al., 2019; Jain et al., 2019; Pajtler et al., 2018; Piunti et al., 2019; Ragazzini et al., 2019). *In vitro* and *in vivo* studies have shown that the C-terminus of EZHIP, which includes a conserved 14 amino-acid motif, is both necessary and sufficient to repress PRC2’s function. This motif, referred to as the K27M-like peptide (KLP), comprises four amino acids, VRMR, that mimic an unmodifiable H3K27M motif (Hübner et al., 2019; Jain et al., 2019; Piunti et al., 2019). Mutating the M (methionine) to a modifiable K (lysine) within the KLP results in wild-type-like levels of H3K27 methylation, suggesting that this EZHIP motif is critical for inhibiting H3K27 methylation, akin to the H3K27M oncohistone mutation, which can no longer be modified by PRC2 (Jain et al., 2019; Piunti et al., 2019). The deletion of *Ezhip* increases global H3K27me2/3 methylation and is correlated with widespread changes in gene expression in cell lines (Piunti et al., 2019; Ragazzini et al., 2019). Conversely, *Ezhip* overexpression results in lower levels of H3K27 methylation, without affecting PRC2 expression, confirming that EZHIP affects PRC2 activity, not its expression. EZHIP also appears to have minimal impact on PRC2 binding to chromatin (Ragazzini et al., 2019) or its initial H3K27 methylation activity. Instead, it suppresses the spreading of PRC2 and H3K27me3 marks across the genome (Jain et al., 2019). Abnormal activation of EZHIP in Posterior fossa type A (PFA) ependymomas, some osteosarcomas, diffuse midline gliomas, and rare non-CNS tumors, is strongly implicated in oncogenesis due to global loss of the repressive histone mark H3K27me3 and epigenetic deregulation of PRC2 targets even in the absence of the H3K27M oncohistone mutation (Cassim et al., 2024; Hübner et al., 2019; Jawhar et al., 2025; Jenseit et al., 2022). A recent study using a *Drosophila* model revealed that human EZHIP is an even more potent inhibitor of PRC2 than the H3K27M oncohistone (Krabbenhoft et al., 2025), suggesting that the EZHIP KLP may have a higher affinity for PRC2 or that EZHIP may have additional sequence features that enhance its inhibitory function.

Previous studies have primarily focused on EZHIP’s role upon aberrant expression in cancer. However, *Ezhip’s* basal expression is primarily restricted to oocytes and (to a lesser extent) testes, with little to no detectable expression in somatic tissues (Ragazzini et al., 2019). Deletion of *Ezhip* in mice results in a global increase of H3K27me2/3 in both spermatogenesis and late-stage oocyte maturation but does not cause gross infertility (Ragazzini et al., 2019). Male knockout mice show a very mild defect in sperm motility. In contrast, female knockout mice show a progressive decline in fertility, with older females having smaller brood sizes and less fit pups, presumably due to excessive H3K27me3 accumulation (Ragazzini et al., 2019). Given its role in PRC2 regulation, it has been proposed that *Ezhip’s* primary function is to ensure that the germline epigenome avoids both over-silencing and inappropriate gene activation, which is crucial for preserving totipotency and for the proper transmission of epigenetic information to the next generation. Nevertheless, *Ezhip’s* biological function remains poorly studied.

Unlike most PRC2 core proteins and cofactors, which are ancient and conserved across most metazoan genomes, EZHIP appears to be mammal-specific (Fischer et al., 2022). Furthermore, EZHIP orthologs seem to show no conservation beyond a 14-amino-acid KLP motif (Jain et al., 2019; Piunti et al., 2019). Yet, functional experiments suggest that residues outside the KLP motif are important for EZHIP function. For example, full-length EZHIP restored PRC2 inhibition activity at lower concentrations than the KLP motif alone (Jain et al., 2019; Ragazzini et al., 2019). Moreover, EZHIP mutants without the KLP motif still retained their ability to interact with EZH2 (Piunti et al., 2019). Lastly, EZHIP has been implicated in DNA damage response in cancer, and this function is independent of the KLP motif (Han et al., 2020). *Ezhip’s* absence outside placental mammals, its rapid sequence divergence except for the KLP motif, and its germline-restricted expression patterns all imply strong, lineage-specific evolutionary pressures.

Here, we use an evolutionary approach to trace the evolutionary origins and cadence of *Ezhip* across 70 mammalian genomes. Consistent with previous results, we find that *Ezhip* arose and has been strictly retained on the X chromosome of placental mammals. Using motif analyses, we demonstrate that, in addition to the previously identified KLP motif, EZHIP encodes seven well-conserved motifs, as well as tandemly repeated sequences that vary in copy number and sequence across different *Ezhip* orthologs. We show that *Ezhip* evolves under diversifying selection in primates. We identify recurrent duplications of *Ezhip* in most mammalian lineages, resulting in *Ezhip* paralogs that have been retained for varying periods. For example, the mouse genome harbors up to 15 young X-linked *Ezhip* paralogs, while two independent duplications of *Ezhip* have been retained in many carnivore and simian primate species. We identify a primate-specific autosomal paralog, *Ezhip2*, which, like ancestral *Ezhip,* evolves under diversifying selection. However, unlike ancestral *Ezhip*, which is primarily expressed in oocytes, the primate paralog *Ezhip2* is expressed exclusively in the testes. Our evolutionary analyses suggest a model in which *Ezhip* arose and diversified through sequence divergence and gene duplication, driven by competition between paternal and maternal genomes to gain control over *in utero* reproduction in placental mammals.

## Results

### *Ezhip* arose and has been strictly retained in placental mammals

**Figure 1.**
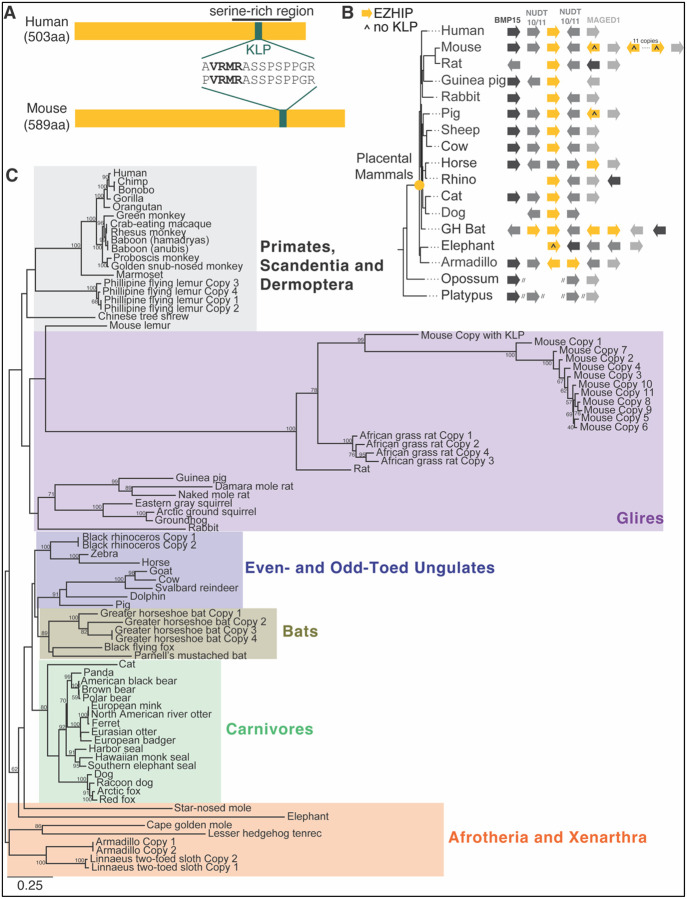
*Ezhip* arose in the last common ancestor of placental mammals. **A.** Schematics of the human and mouse ancestral EZHIP highlight previously identified domains, a serine-rich region (black line) and a highly conserved H3K27M-like peptide (KLP) (green), annotated as previously described (Hübner et al., 2019; Jain et al., 2019; Piunti et al., 2019; Ragazzini et al., 2019). The KLP sequences for mouse and human are shown. The lengths of human and mouse EZHIP are denoted on the left side of the schematic. **B.** A schematic representation of *EZHIP* genes (yellow) in their genomic neighborhood (shades of grey) is shown beside a mammalian species tree (GH Bat stands for greater horseshoe bat). Genes in the syntenic neighborhood near *EZHIP* are represented with various shades of gray arrows: dark gray (*bmp15*), medium gray (*nudt10/11*), and light gray (*maged1*). *EZHIP* genes without KLP are denoted with a caret (^) symbol. A yellow dot on the species tree indicates the origin of *EZHIP* in placental mammals. Homology searches for *maged1* in a few species (horse, cat, and elephant) yielded many matches, suggesting *maged1* may have been duplicated in these species. In these cases, we have annotated the match with the closest genomic location to *EZHIP*. Ends of genomic scaffolds are represented with two slashes. **C.** A maximum-likelihood protein phylogeny of syntenic EZHIP copies with complete ORFs across 64 representative mammalian species. Colored boxes in the phylogeny indicate different mammalian lineages – Primates (gray), Glires (purple), Carnivores (green), Ungulates (blue), Bats (brown), and Afrotheria and Xenarthra (orange). Bootstrap values greater than 50 are shown alongside the node they represent. The bottom-left scale bar below the phylogeny represents a time of 0.25 substitutions per site.

The single-exon *Ezhip* gene is located on the X chromosome in the human genome, where it encodes a 503-amino-acid EZHIP protein (Figure 1A). The mouse X chromosome also encodes an *Ezhip* ortholog, which encodes a 589-amino-acid protein. Previous studies have concluded that there is only limited sequence similarity between these two orthologs, with the histone mimic-like KLP motif being the only common feature. To systematically identify *Ezhip* homologs in mammals, we interrogated genome assemblies from 69 placental mammals, one marsupial (opossum), and one monotreme (platypus). We used the human EZHIP sequence as a query to perform homology-based searches against each genome (see Methods). This approach successfully identified *Ezhip* homologs in most placental mammals but not in marsupials or monotremes. Due to substantial divergence from human EZHIP, we were unable to identify *Ezhip* homologs in some placental mammal species, especially in rodents, despite the mouse genome previously being shown to encode *Ezhip*. Therefore, we performed iterative homology searches, using EZHIP sequences from more closely related species as queries, to identify homologs across all queried placental mammals (Supplementary Table S1).

To confirm putative *Ezhip* orthologs, we relied on analyses of shared synteny (*i.e.,* conserved genomic neighborhoods) in representative species. To this end, we used genes flanking *Ezhip* on the X chromosome, which are deeply conserved across mammals, to identify the syntenic ancestral locus. We identified the *Ezhip* gene at this shared syntenic location in representative mammals from different lineages (Figure 1B). This allowed us to conclude that *EZHIP* has been conserved at the shared syntenic location on the X chromosome of placental mammals. We could not fully reconstruct this syntenic location in platypus or opossum. In the platypus genome, we identified the flanking genes on completely distinct scaffolds due to incomplete genome assembly. In the opossum genome, the flanking genes were fragmented between two chromosomes– NUDT10/11 and MAGED1 were on chromosome X, while BMP15 was found on chromosome 8. While we were still able to identify the flanking genes, we did not find *Ezhip*. We further queried the KLP motif against these genomes and were still unable to identify even this well-conserved EZHIP fragment. Together, this confirms that EZHIP arose in the last common ancestor of placental mammals.

In almost all placental mammals, we identified the KLP motif within their respective EZHIP orthologs. However, in elephants (both *Loxodonta africana* and *Elephas maximus*), the KLP has been lost from their EZHIP orthologs (caret symbol in Figure 1B, Supplementary Figure S1). Intrigued by this single instance of loss of a near-universal EZHIP motif, we used the 14-amino-acid KLP motif to search all Ensembl-predicted proteins in the elephant genome using an alignment-independent MAST search (Bailey & Gribskov, 2000) and still failed to detect the KLP motif. Thus, this motif appears to be missing entirely from the elephant genome. This seems to be unique to elephants, as other related Afrotherian species, such as the cape golden mole and lesser hedgehog tenrec, still have an intact KLP in their EZHIP (Supplementary Figure S1).

Most mammalian *Ezhip* orthologs have a well-conserved start codon. However, a multiple alignment unexpectedly revealed recurrent mutations at the annotated *Ezhip* start codon in a few cases (Supplementary Figure S2). For example, *Ezhip* orthologs from gorilla, *Cercopithecinae* (Old World monkeys), and *Platyrrhini* (New World monkeys) have a mutation at the position corresponding to the annotated start codon of human EZHIP (Supplementary Figure S2A). We hypothesize that monkeys and apes instead use a methionine five amino acids downstream of the annotated start, which is well conserved across primates. Dolphin *Ezhip* also has a mutation of the annotated start codon. Furthermore, several other *Ezhip orthologs* (elephant, tenrec, cat, and cow) encode proteins with a divergent N-terminal sequence, in which the start codon cannot be readily aligned with those of other placental mammals (Supplementary Figure S2B). Based on these findings, we infer that some placental mammal *Ezhip* orthologs have evolved an alternate start codon.

Using the multiple alignment of EZHIP sequences found within the syntenic location, we carried out phylogenetic analyses using PhyML (Guindon et al., 2010; Guindon & Gascuel, 2003) (Figure 1C, Supplementary Data S1). We found that their phylogenetic relationships matched our expectations based on mammalian phylogeny. Our analyses thus confirm previous conclusions that *EZHIP* originated on the X chromosome in the last common ancestor of placental mammals (Ragazzini et al., 2019). The fact that *EZHIP* is strictly retained in placental mammals suggests it may be essential for mammalian reproduction or development.

### *Ezhip* orthologs encode eight well-conserved motifs and independently arising tandem repeats

Previous studies have primarily focused on human and mouse EZHIP orthologs, which were thought to share only a 14-amino-acid KLP motif that includes the H3K27M-like VRMR (Figure 1A). Subsequent studies have noted the presence of other protein domains, such as a serine-rich region (Pajtler et al., 2018) or repetitive sequences (Piunti et al., 2019), but these were considered idiosyncratic occurrences; most of the ~500-amino-acid EZHIP protein remained uncharacterized.

**Figure 2.**
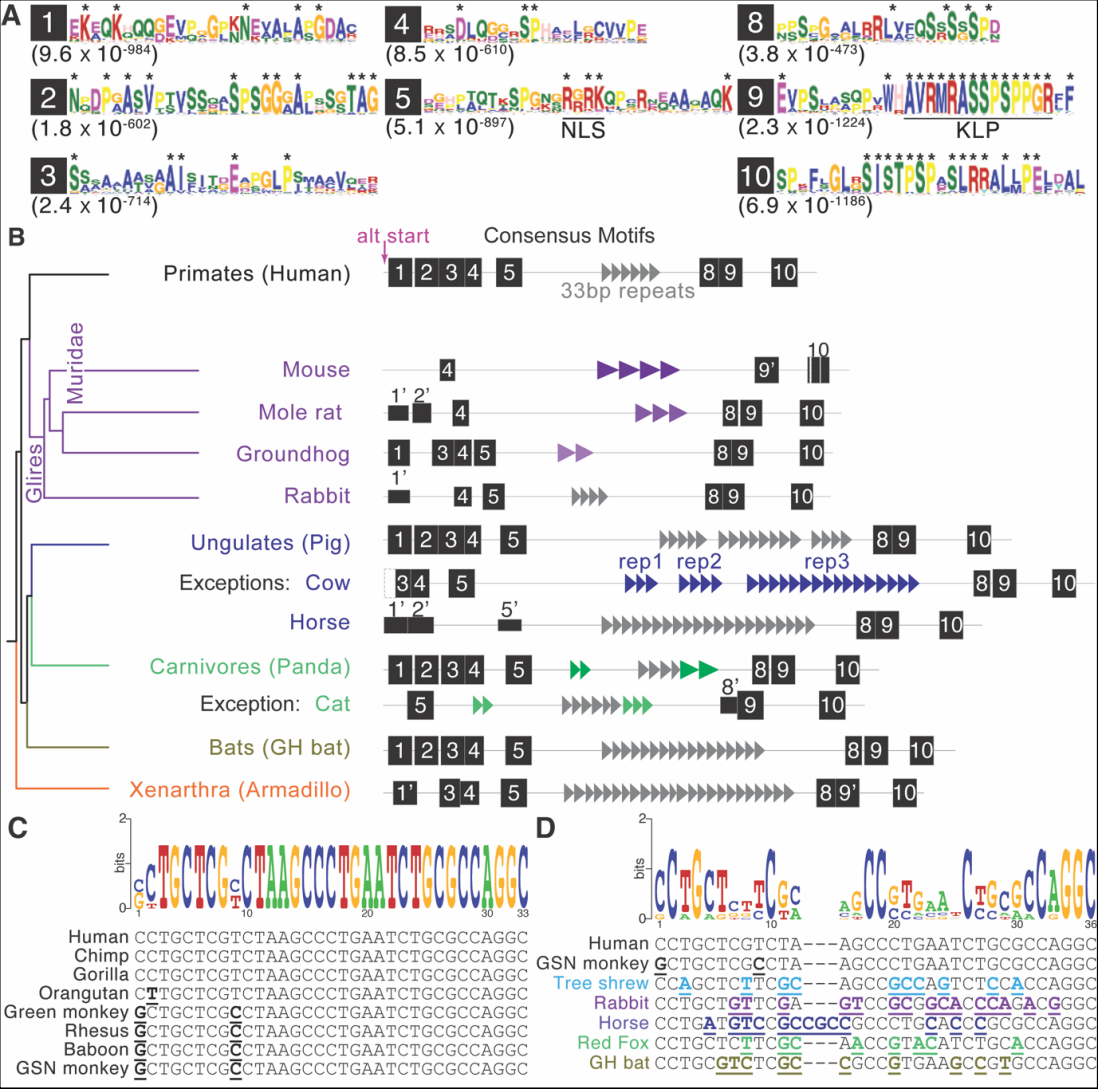
EZHIP has eight conserved motifs and tandem repeats across placental mammals. **A.** Protein logo plots of eight motifs in syntenic EZHIP genes discovered using MEME analyses (Bailey et al., 2015). The e-values are noted below each motif. Asterisks indicate residues that are present in >90% of mammalian species included in the analyses, as evidenced by their height. A Nuclear Localization Signal (NLS) we predicted by PSORT analyses (Nakai & Horton, 1999) and the previously identified H3K27-Like Motif (KLP) with the oncohistone-like VRMR motif in motif 5 and motif 9, respectively, are indicated. **B.** A simplified mammalian species tree (colored as in Fig. 1C) is shown alongside a representative protein schematic with identified motifs (black filled boxes with numbers 1–5 and 8–10). The representative species for each clade is specified in parentheses, and rare exceptions that deviate from the observed pattern are indicated below. GH bats refer to the greater horseshoe bat. All mammalian species analyzed are shown in Supplementary Figure S3. The pink arrow indicates the alternate start site in primates. Motif heights represent the significance of a motif site within the sequence, with taller motifs representing more statistically significant sites. In some instances, motifs were only identified by one program (MAST or MEME), as indicated with an apostrophe (‘). Cases where part of a motif could be identified are represented as boxes with dashed lines (e.g., motif 3 in cow or motif 10 in mouse). See Methods for details. The only mammalian lineage that showed a high divergence in motif retention was Glires, for which multiple species are shown. Motifs 6 and 7 were highly repetitive (Supplementary Figure S3), so we used Tandem Repeat Finder (TRF) to detect repeats in EZHIP. Most lineages had varying numbers of a common 33bp tandem repeat (gray triangles), and in rare instances, we found lineage-specific repeats (white, green, blue, or purple repeats). In some cases, such as cow, 3 different repeat types can be found. Within glires, shades of purple represent poor homology of repeats, suggesting species-specific repeats. Motifs are drawn to scale, but triangles indicating repeats are not to scale, with each grey triangle representing 33 bp. **C, D.** Alignment of the common tandem repeat in simian primates (C) and in a wider set of mammals (D). Top, DNA logo plots, where taller nucleotides indicate higher conservation. Bottom, nucleotide alignments, where nucleotides that differ from the human tandem repeat are bolded and underlined in their lineage-specific colors. GSN monkey refers to the golden snub-nosed monkey.

We leveraged our comprehensive identification of EZHIP orthologs across placental mammals to uncover motifs common to most orthologs to gain insight into the functional constraints that have shaped EZHIP function and divergence across mammals. Rather than rely on multiple alignments, whose accuracy could be affected by high sequence divergence or length differences, we instead relied on *de novo* identification of conserved motifs across using the MEME suite (Bailey et al., 2015), as previously described (Caro et al., 2022; Kursel & Malik, 2017) (see Methods). Using the MEME suite, we identified ten conserved motifs at high statistical significance in the majority of EZHIP orthologs from placental mammals (Figure 2A). The newly identified motifs include residues that are near universally conserved in mammalian species (asterisks in Figure 2A, Supplementary Data S2).

We mapped the occurrence of the motifs onto each of the orthologs using the MAST software within the MEME suite (Figure 2B, Supplementary Figure S3). We numbered these motifs sequentially from N- to C-terminus. Based on this numbering scheme, the previously identified KLP motif falls within motif 9. As expected, this motif had the highest statistical significance of all ten motifs; it is present and nearly identical across all orthologs, except elephant EZHIP, where it has unexpectedly been lost (Figure 1B, Supplementary Figure S3). The MAST analysis revealed that, in addition to KLP (within motif 9), several additional motifs are broadly conserved across most mammalian EZHIP orthologs (Figure 2B, Supplementary Figure S3). Using PSORT analyses (Nakai & Horton, 1999), we also identified a putative nuclear localization signal (NLS) within motif 5 (Figure 2A).

Among the ten motifs, MAST analysis found two motifs (6 and 7) more than once in each ortholog (Supplementary Figure S3). In contrast, the other eight identified motifs (motifs 1–5, 8–10) are represented only once in most EZHIP orthologs. These eight motifs include residues that are highly conserved across mammals (indicated by asterisks). However, there were some striking exceptions to the near-universal conservation of these eight EZHIP motifs, most notably among Glires (including rodents) (Figure 2B, Supplementary Figure S3). Most EZHIP orthologs in *Glires* retain motifs 4, 5, 9, and 10, but with much lower statistical support than in other mammals. Motifs 1, 3, 5, and 8, which are otherwise prevalent across other mammalian EZHIP orthologs, are much less conserved in *Glires* and are entirely absent in *Muridae*. This low retention of otherwise well-conserved motifs within *Muridae* is likely due to high EZHIP divergence within this lineage, as evidenced by their long branch lengths for orthologs in the phylogenetic analyses (Figure 1C). Furthermore, we were unable to identify robust rodent-specific motifs when we limited our analyses to rodent EZHIP orthologs. *Glires* and *Muridae* are the most striking examples of EZHIP motif loss, but such motif loss also occurs idiosyncratically in other lineages (Figure 2B, Supplementary Figure S3). N-terminal motifs are more likely to be lost than C-terminal motifs, consistent with previous studies that showed that the C-terminal EZHIP domain encompassing the KLP motif is necessary and can be sufficient for EZHIP’s PRC2 inhibitory function (Hübner et al., 2019; Piunti et al., 2019).

Our finding that motifs 6 and 7 were repetitive within most orthologs (Supplementary Figure S3) suggested they might be part of a repetitive region in EZHIP. To formally address this possibility, we used Tandem Repeat Finder (TRF) (Benson, 1999) to identify tandem repeat sequences present in EZHIP sequences from ~50 mammalian species. We then manually curated the results of this analysis to determine the smallest repeat unit and mapped the number of repeats within representative EZHIP orthologs (Figure 2B). Repeat units vary dramatically across EZHIP orthologs, ranging from as few as 3 copies in bats and some carnivores to as many as 21 copies in ungulates (Figure 2B). Using the Genome Aggregation Database (gnomAD) (Karczewski et al., 2020) we find that the number of EZHIP tandem repeats varies even within humans. Using iterative sequence alignments within a lineage (*e.g.,* among carnivores) and between lineages (*e.g.,* between carnivores and primates), we identified patterns of common and lineage-specific repeats. Based on these comparisons, we infer that most EZHIP orthologs contain a related ~33 bp repeat, suggesting this was a feature of ancestral EZHIP since its origin. For example, we can identify related tandem repeats in primates, ungulates, some carnivores (such as *Canidae*), and bats (Figure 2B). Although we were unable to detect this repeat in rodents, we could detect it in rabbit EZHIP. The repeat unit is relatively well-conserved both within a species and across lineages (Figure 2D, Supplementary Figure S4), with >95% pairwise identity within simian primates (Figure 2C), and >64% pairwise identity between lineages (Figure 2D). In addition to the ~33 bp repeat present across most mammalian EZHIP proteins, we found that some lineages have also evolved ‘new’ lineage- or even species-specific tandem repeats (represented in lineage-specific colors, Figure 2B), which show sequence, length, and copy-number divergence. For example, although the ancestral 33 bp repeat is present in *Canidae*, other carnivores have lost this ancestral repeat but gained three new repeats (33 bp, 39 bp, and 66 bp). Similarly, while most ungulates retained the ancestral 33 bp repeat, species like cow lost this ancestral repeat and gained three new repeat families (each 36 bp long). Lastly, each of the three rodent species we analyzed lost the ancestral 33 bp repeat but gained new repeats, each different in length and sequence (Figure 2B). Given the dynamics of tandem repeats and the long evolutionary timescale, we cannot rule out the possibility that these new repeats are themselves derived from the ancestral 33 bp repeat. Nevertheless, as with the seven new motifs we have identified, tandem repeats appear to have been a feature of EZHIP orthologs throughout placental mammal evolution.

### *Ezhip* has evolved under diversifying selection

**Figure 3.**
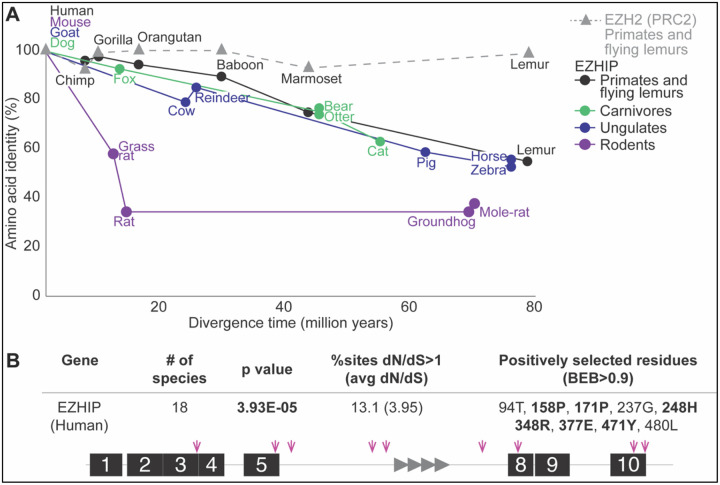
EZHIP shows signatures of rapid evolution. **A.** Divergence time (in million years) is plotted against amino acid identity (%). Amino acid identity of EZHIP (circle) from different mammalian lineages (colored as in 1C), where selected orthologs are compared to a representative species (human for primates, mouse for rodents, goat for ungulates, and dog for carnivores). Amino acid identity of EZH2 (gray triangle), a component of the PRC2 complex, is compared across primates against human. **B.** PAML and FUBAR analyses were used to look for site-specific positive selection. Top, p-value from PAML’s Model 8 versus Model 8a comparison (Yang, 1997), with percentage of sites with dN/dS>1 and the estimated average dN/dS. Positively selected sites identified by PAML (M8 BEB>0.9) are shown with those also found with FUBAR (Murrell et al., 2013) highlighted in boldface. Bottom, a motif schematic of EZHIP is shown, with pink arrows indicating the location of residues that are under positive selection. Also see Supplementary Figures S5 and Supplementary Table S3.

Given its strict retention, we infer that *Ezhip* performs a vital function in the germline tissues of all placental mammals. However, our analyses have also revealed an apparent disparity among placental mammalian lineages in the evolutionary divergence of EZHIP, with some lineages exhibiting long branches (Figure 1C) or lacking ancestral protein motifs (Figure 2, Supplementary Figure S3). To investigate this disparity further, we compared the rate of EZHIP protein divergence across different mammalian lineages, including primates, rodents, carnivores, and ungulates, spanning nearly 70 million years of evolution in each lineage (Figure 3A, Supplementary Table S2). For our analyses, we measured the pairwise identity of EZHIP orthologs relative to a ‘reference’ ortholog within that clade. For example, we compared the pairwise identities of various primate EZHIP orthologs to human EZHIP. We then compared this protein divergence to the estimated species divergence times obtained from TimeTree (Hedges et al., 2015). To be conservative and consistent, we chose the most conserved ortholog when multiple paralogs were found in the shared syntenic location. We carried out similar comparisons in rodents (compared to mouse EZHIP), ungulates (compared to goat EZHIP), and carnivores (compared to dog EZHIP). Our analyses revealed that EZHIP orthologs in primates, ungulates, and carnivores exhibit accelerated divergence, with as little as ~60% conservation among orthologs. This is in stark contrast to EZH2, a PRC2 component that interacts with EZHIP, which is >90% identical across primates. Rodent EZHIP orthologs show an even more accelerated divergence, with only 40% identity within a 20 MY time span (Figure 3A). Our results are consistent with our EZHIP phylogeny and motif analyses (Figure 1C and Figure 2B) and with previous evolutionary analyses that highlight the much faster rate of divergence in rodent genomes (Wu & Li, 1985).

A high degree of EZHIP divergence across mammals could indicate relaxed constraint, although this is unlikely given *Ezhip’s* strict retention in placental mammals. We examined selective constraints acting on *Ezhip* by comparing the normalized ratio of amino-acid-altering changes (non-synonymous, dN) to amino-acid-preserving changes (synonymous, dS). Neutrally evolving sequences are expected to have dN/dS close to 1, whereas sequences under selective constraint (purifying selection) are expected to have dN/dS < 1. Using a set of mammalian species from the lineages selected above (Figure 3A), we compared the relative likelihoods of codon models assuming neutral versus non-neutral evolution. Specifically, we used PAML’s codeml program (Yang, 1997) to estimate the likelihood of a simple evolutionary model (Model 0), where all sequences and all codons are assumed to have the same dN/dS ratio. We compared model 0, with the likelihood of dN/dS fixed at 1 (neutral, as expected for a pseudogene), to that of dN/dS estimated from the alignment (~0.85). This test revealed evidence for purifying selection on *Ezhip* across mammals (Supplementary Table S3, Supplementary Data S3), formally ruling out the possibility that the observed high protein divergence is due to relaxed constraint.

Despite this overall signature of purifying selection, a subset of *Ezhip* codons could evolve under positive selection (dN/dS >1). To investigate this possibility, we analyzed sequences from simian primates; this clade has an ideal level of evolutionary divergence for codon-by-codon analyses (McBee et al., 2015). We analyzed intact *Ezhip* sequences from 18 simian primates, removing repetitive regions because they are difficult to align confidently (Supplementary Table S4, Supplementary Data S4). We assessed codons undergoing positive selection through maximum likelihood analyses using PAML (Yang, 1997) and FUBAR (HyPhy package (Murrell et al., 2013; Pond et al., 2005)). PAML analyses revealed that 13.1% of EZHIP sites are estimated to evolve with an average dN/dS of 7.01 (Figure 3B, Supplementary Figure S5). A subset of these sites met the Bayes Empirical Bayes (BEB) statistical threshold of 90%. FUBAR analyses identified nearly the same sites, and some additional sites under positive selection (Figure 3B, Supplementary Table S4). About half of the positively selected residues in EZHIP are found within previously identified protein motifs that are well aligned across primates, ruling out the possibility that primate *Ezhip’s* positive selection is an artifact caused by poor alignments. Instead, our findings show that *Ezhip* is undergoing purifying selection at the whole-gene level, but that a subset of sites has been subject to diversifying (positive) selection, at least in primates.

### *Ezhip* has recurrently duplicated in placental mammals

Previous studies have focused exclusively on *Ezhip* orthologs located in the same syntenic region. However, our analyses also identified multiple within-species duplications of *Ezhip*, creating paralogous copies (Supplementary Figure S6). Using protein sequence alignments of all identified *Ezhip* orthologs and paralogs (Supplementary Table S1), we manually curated all paralogs as being either incomplete due to gaps in genome assembly (‘*i*’, Supplementary Figure S6), or pseudogenes with obvious disruptions in the open-reading frame (boxes containing crosses, Supplementary Figure S6), or lacking a KLP motif (caret, Supplementary Figure S6). We note that incomplete *Ezhip* paralogs or those lacking a KLP motif may still be selectively retained if they encode functional proteins (*e.g.,* elephant *Ezhip*), although an intact KLP motif has previously been shown to be essential for PRC2 regulation (Jain et al., 2019; Piunti et al., 2019).

In some genomes, we found multiple *Ezhip* homologs at the shared syntenic location on the X chromosome in the Philippine flying lemur, the African grass rat, the greater horseshoe bat, the rhino, the armadillo, and the sloth genomes (Supplementary Figure S6). Other *Ezhip* paralogs are found at different locations on sex chromosomes (blue boxes), on autosomes (pink boxes), or in unmapped locations (black boxes). The most spectacular example of *Ezhip* duplications is seen in the mouse genome, which encodes 15 X-chromosomal *Ezhip* paralogs (Figure 1B, Supplementary Figure S6, Supplementary Table S1), four of which appear to be pseudogenes lacking an intact open reading frame. The remaining 11 mouse *Ezhip* paralogs appear to be intact but lack an intact KLP motif (Figure 1B (caret symbol); Supplementary Figure S7).

Phylogenetic analyses of EZHIP and its paralogs using maximum likelihood methods (Supplementary Figure S8, Supplementary Data S5) revealed that most paralogs are species-specific, *i.e.,* they are more closely related to the ancestral EZHIP orthologs from their own species than to EZHIP genes from other species. However, not all EZHIP duplication events are young. We identified at least two instances in which EZHIP paralogs have been selectively retained for long periods. The first of these is in carnivores, where an *Ezhip* paralog appears to have been retained for 60 million years (Supplementary Figure S8). We traced this *Ezhip* paralog to a single X-chromosomal duplication event in the last common ancestor of *Ursidae, Mustelidae, and Pinnipedia* (Supplementary Figure S9). This *Ezhip* paralog in the dog genome has undergone pseudogenization and is absent from cat genomes, even at syntenic locations. It has undergone further duplication in *Ursidae, Mustelidae, and Pinnipedia,* resulting in additional intact or pseudogenized copies that group together in phylogenetic analyses (Supplementary Figure S6, S8). Most of these paralogs lack an intact KLP motif, suggesting they may have lost their PRC2-inhibitory functions.

**Figure 4.**
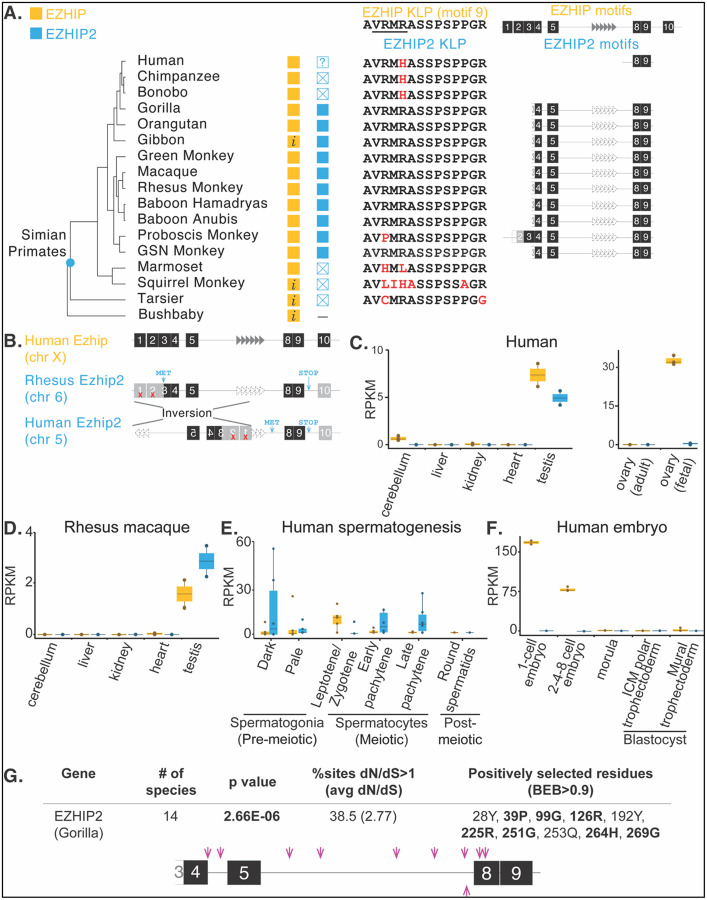
A primate-specific paralog, EZHIP2, has testis-enriched expression and retains some motif conservation. **A.** Left, EZHIP (yellow boxes) and EZHIP2 (blue boxes) are illustrated alongside a primate species tree. Boxes containing an “X” represent putative pseudogenes, “*i*” represents incomplete sequences due to gaps in genome assembly, and “?” represents a shortened open reading frame (see B). The duplicate was not identified in bushbaby through homology or synteny analyses, suggesting *Ezhip2* arose in *Haplorhini* (blue dot in species tree). Middle, the KLP sequence with the oncohistone-mimic region underlined from human ancestral EZHIP (top) is compared to the KLP sequence across EZHIP2, with changes relative to ancestral EZHIP in red. Right, motif schematics for human EZHIP and possibly intact primate EZHIP2 sequences (also see Supplementary Figure S10). Empty boxes indicate incomplete motifs, and grey boxes indicate the presence of gaps in the sequence. Dashed triangles indicate divergence of tandem repeat regions that are no longer identified as repeats by Tandem Repeat Finder. **B.** EZHIP2 may have arisen via retrotransposition and has undergone an inversion in humans. A genomic arrangement of *Ezhip* compared to rhesus *Ezhip2*, which encodes a slightly shorter protein (316 amino acids) and human *Ezhip2*, which shows an inversion, but which could still encode an 88 amino acid protein that includes the KLP, is shown. EZHIP is on a sex chromosome, but EZHIP2 is on an autosome at the same syntenic location in the analyzed species. The red X indicates nonsense mutations in motifs 1 and 2. A methionine was gained in motif 3, and a premature stop codon after motif 9 results in a shorter open reading frame for most primate EZHIP2. **C-F.** *EZHIP2* is exclusively expressed in testes. Public RNA-seq data (Brawand et al., 2011; Hendrickson et al., 2017; Jan et al., 2017; Williams et al., 2015) from somatic tissues (cerebellum, liver, kidney, and heart) and germline tissues (ovaries and testes) were analyzed for the human (C) and rhesus macaque (D) for EZHIP and EZHIP2. Expression (RPKM or reads per kilobase per million reads sequenced) is plotted for each tissue, with standard error bars. E, F. Expression of *EZHIP* paralogs across stages of human spermatogenesis and embryonic development is plotted as in C. **G.** *EZHIP2* is rapidly evolving in simian primates. Top, p-values, percentage of sites with dN/dS>1, and the estimated average dN/dS from PAML are shown. Positively selected sites identified by PAML are indicated, with those also identified by FUBAR highlighted in boldface. Bottom, a motif schematic of gorilla EZHIP2 is shown, with pink arrows indicating the location of residues that are under positive selection. Also see Supplementary Figures S11 and Supplementary Table S4.

A second ancient *Ezhip* duplication, which we named *Ezhip*2, arose once on an autosome (human chromosome V) in the last common ancestor of *Haplorhini* (simian primates and tarsier) and appears to have been largely retained for ~40 million years (Figure 4A, Supplementary Figure S8). *Ezhip* and *Ezhip*2 share no syntenic regions, suggesting that *Ezhip2* may have arisen via a retrotransposition event. *Ezhip*2 appears to be retained in simian primates except in two instances: bonobo/chimpanzee and marmoset/squirrel monkey, which have acquired pseudogenizing mutations and lack an intact KLP motif. Otherwise, *Ezhip2* genes from Old World monkeys and hominoids encode motifs 4–5 and 7–9, including a well-conserved KLP motif, but lack any obvious tandem repeats (Supplementary Figure S10). The status of human *Ezhip*2 remains less certain. The N-terminal region of human *Ezhip*2 underwent an inversion, leaving a much shorter open reading frame (88 amino acids) that only encodes motifs 8 and 9, including an intact KLP motif (Figure 4B). Although the (inverted) N-terminal segments have acquired pseudogenizing nonsense mutations, human *Ezhip*2 still seems capable of encoding an intact, albeit much shorter, open reading frame using a novel start codon upstream of motif 8. Despite its truncated nature, human EZHIP2 might still be capable of inhibiting PRC2, as previous experiments showed that an intact KLP motif and surrounding segments can be sufficient for H3K27me inhibition (Jain et al., 2019; Piunti et al., 2019).

To further assess whether *Ezhip*2 could be functional, we analyzed the expression of *Ezhip* and *Ezhip*2 using public RNA-seq data (Supplementary Table S5) from human (Figure 4C) and rhesus macaque (Figure 4D). As in previous work (Ragazzini et al., 2019), we found that *Ezhip* is undetectable in most somatic tissues. This was also true for *Ezhip2*. The only exception was that, unlike prior work, we detected a low level of *Ezhip* expression, but not *Ezhip2* expression, in the cerebellum, a tissue where *Ezhip* is often mutated or overexpressed in PFA ependymomas (Hübner et al., 2019). Consistent with previous work, we find that *Ezhip* expression is highest in reproductive tissues, both testis and ovaries, with higher expression in fetal ovaries than in adult ovaries (Figure 4C) (Ragazzini et al., 2019). In contrast, *Ezhip2* expression appears to be limited to the testes. This pattern of *Ezhip2* expression in testes is also recapitulated in rhesus macaques, which encode full-length *Ezhip2* (Figure 4D). Both *Ezhip* and *Ezhip2* are expressed early in human spermatogenesis (Figure 4E), with no expression detected in sperm post-meiosis. *Ezhip2* expression increases slowly, reaching its highest level in pre-meiotic sperm, then decreases during meiosis. Lastly, while *Ezhip* is highly expressed in the embryo, we do not detect *Ezhip2* (Figure 4F). Together, these differential expression patterns suggest that *Ezhip* and *Ezhip2* may have evolved unique expression patterns and potentially, non-redundant functions in the male germline in most simian primates.

Another approach to assess whether *Ezhip2* is functional is to examine whether it evolves under selective constraints, like *Ezhip*. We analyzed full-length *Ezhip2* sequences from 14 non-human simian primates, excluding all primates with obvious pseudogenizing mutations and humans, which encode a much-truncated *Ezhip2* (Supplementary Table S4, Supplementary Data S6). Our analyses allow us to reject the hypothesis that *Ezhip2* evolves neutrally. Using PAML analyses, we inferred that *Ezhip2* evolves with an overall dN/dS of 1.35 with a higher likelihood (p-value=0.02) than the null hypothesis of neutral evolution (dN/dS =1). Consistent with the whole-gene estimate of diversifying selection, our codeml analyses also found that 38.5% of *Ezhip2* codons evolve with a dN/dS of 2.77 (Supplementary Table S4). Several sites meet the Bayes Empirical Bayes threshold of 0.9. FUBAR analyses also identified these sites, along with additional sites that likely evolved under positive selection (Figure 4G, Supplementary Table S4, Supplementary Figure S11). Our selection analyses find evidence that *Ezhip* paralogs have undergone recurrent innovation in placental mammals, both through site-specific positive selection (in primates) and recurrent whole-gene duplications. Thus, despite being strictly retained in placental mammals, *Ezhip* and its paralogs exhibit signatures of participation in biological processes that require constant innovation.

## Discussion

In this study, we have revisited the earlier exciting discovery of EZHIP, a ‘histone mimic’ based negative regulator of PRC2 (Hübner et al., 2019; Jain et al., 2019; Piunti et al., 2019; Ragazzini et al., 2019), by characterizing its evolution across 70 mammalian species to understand its functional significance. Our study reveals that *Ezhip* originated on the X chromosome in the last common ancestor of placental mammals and has been strictly retained since its origin. Beyond the previously identified KLP motif, we identified seven additional conserved motifs, many of which are near universally retained across mammals, and lineage-specific tandem repeats. Most strikingly, *Ezhip* exhibits strong signatures of genetic innovation, undergoing both diversifying selection in primates and recurrent duplications and post-duplication losses across mammalian lineages. *Ezhip* paralogs include 11 X-linked paralogs in mice and two independent autosomal duplications retained for long periods in carnivore and primate evolution. Primate *Ezhip2* also evolved under positive selection and shows testis-specific expression. Thus, although the strict retention of *EZHIP* in placental mammals suggests an important conserved function, our discovery of constant innovation implies that *Ezhip* and its paralogs are also subject to changing selective pressures that require ongoing adaptation. These recurrent signatures of innovation suggest its involvement in genetic conflict and might provide vital clues into *Ezhip’s* function.

*Ezhip* arose in placental mammals, whereas the PRC2 complex may date back close to the origin of eukaryotes (de Potter et al., 2023; Sharaf et al., 2022; Vijayanathan et al., 2022). The three primary functions carried out by PRC2 in multicellular eukaryotes are developmental transitions (Laugesen et al., 2019; Yu et al., 2019), transposon defense (Hisanaga et al., 2023; Shaver et al., 2010), and genomic imprinting (Yu et al., 2019). Of these three roles, genome imprinting is the only function that occurs exclusively in placental mammals. Genetic imprinting is an epigenetic process in which gene expression is determined based on whether a gene is inherited from the mother or the father. Pioneering work from Robert Trivers and David Haig has suggested that this process may have evolved in placental mammals due to an epigenetic tug-of-war between the paternal and maternal genomes over resource allocation to the embryo *in utero* (Constância et al., 2004). Given its recurrent innovation, evolutionary origin in placental mammals, and germline-enriched expression, we hypothesize that *Ezhip* arose and continues to rapidly evolve to regulate genome imprinting in placental mammals. Indeed, a recent study directly implicates EZHIP in imprinting function in mice (Zeng et al., 2025).

The evolutionary characteristics of *Ezhip* – placental mammal-specific origin, X-chromosomal localization, germline-specific expression, rapid sequence divergence, and dynamic gene duplication – are highly reminiscent of the short histone H2A histone variants that arose and proliferated on the X chromosome of placental mammals (Molaro et al., 2018). We recently demonstrated that one of the short H2A histone variants, H2A.B, functions as a biparental-effect gene, with both paternal and maternal contributions synergistically promoting embryo viability and *in utero* growth (Molaro et al., 2020). Like short histone H2A variants, particularly H2A.B, *Ezhip* exhibits evolutionary signatures characteristic of genes mediating genetic conflict between maternal and paternal genomes. Both short H2A variants and *Ezhip* originated exclusively in placental mammals, coincident with the evolution of invasive placentation, and both are X-chromosomal, which could facilitate the rapid evolution of parent-of-origin effects that mediate conflicts over resource allocation to offspring through hemizygous selection in males. The recurrent duplication and divergence of *Ezhip* across mammalian lineages (including 11 X-chromosomal EZHIP paralogs in mice) mirrors the extraordinary evolutionary dynamism of short H2A variants (including up to 20 H2A.L genes in mice (Molaro et al., 2020)). Finally, *Ezhip’s* accelerated evolution parallels that of the H2A.B and H2A.P variants, suggesting that *Ezhip* participates in a molecular arms race, as expected for genes mediating genetic conflict, where paternal and maternal contributions have antagonistic optima. Imprinted loci vary between mammalian species and even between closely related primate species (Ahn et al., 2025; Bogutz et al., 2019; Cheong et al., 2015; Hu et al., 2023). Therefore, it is tempting to speculate that the evolutionary innovation of *Ezhip* may facilitate differential gene regulation of imprinted loci in placental mammals.

The multiple parallels between short histone H2A variants, such as H2A.B, and *Ezhip* further suggest that *Ezhip’s* primary function might be to help mediate the interparental conflict over embryonic development *in utero* in placental mammals. *Ezhip’s* germline-restricted expression and its role in regulating H3K27me3, which marks imprinted genes controlling growth and resource allocation, could position it directly at the interface between parental genomes. Our findings that the primate-specific *Ezhip2* paralog is expressed exclusively in the male germline, whereas *Ezhip* is expressed in both ovary and testis, suggest these paralogs may have functionally diverged and now carry out distinct roles in mediating paternal versus maternal influences on offspring development, with paternally expressed genes predicted to favor increased maternal investment and maternally expressed genes predicted to balance investment across all offspring.

Our study also has implications for *Ezhip’s* multi-faceted functions. Systematic truncations of EZHIP expressed in human cells reveal that while the KLP motif is necessary, it is not sufficient to inhibit H3K27 methylation (Hübner et al., 2019; Jain et al., 2019; Piunti et al., 2019) or to bind PRC2 (Jain et al., 2019; Piunti et al., 2019). Together, these data suggest that EZHIP must have additional motifs that facilitate its only well-characterized function: binding and inhibition of PRC2. Yet, previous studies had focused almost exclusively on the conservation of the KLP motif. Our study reveals several additional ancestrally conserved motifs, whose conservation implies they also likely participate in EZHIP’s role in regulating PRC2. This may occur either by increasing the binding avidity to the PRC2 complex or by altering its localization to different genomic loci, potentially changing the landscape of H3K27me3 modifications across germline genomes. We posit that over-reliance on the highly divergent mouse EZHIP, one of the earliest EZHIP orthologs identified, may have obscured the identification of conserved motifs. This highlights the power of *de novo* motif identification over alignment-based methods to identify regions of homology and functional constraint in highly divergent proteins such as EZHIP. We predict that mutating these well-conserved motifs may reveal key insights into EZHIP’s functions in future studies. The importance of these motifs is especially highlighted by the continued retention of the full-length *Ezhip* gene in elephant species, despite its loss of the KLP motif. Moreover, aberrant *Ezhip* expression in PFA ependymomas has recently been shown to suppress homologous recombination-mediated DNA repair independent of the KLP motif, suggesting that EZHIP may modulate the DNA damage response (Han et al., 2020). It is exciting to speculate that *Ezhip* orthologs or paralogs may have functions independent of PRC2-inhibition that contribute to their evolutionary retention.

Our study also has implications for previous and future genetic studies of *Ezhip* function. The best-known system in which *Ezhip’s* canonical function has been elucidated is in mice (Ragazzini et al., 2019), where *Ezhip’s* knockout phenotypes were surprisingly modest given the strict retention of *Ezhip* in placental mammals: no effect on male fertility and an age-dependent decline in female fertility. Our finding that the mouse genome encodes at least 11 additional intact *EZHIP* paralogs (lacking the KLP motif) suggests that single ortholog knockouts may be insufficient to study the consequences of a complete loss of *Ezhip* activity in mice. Future genetic perturbations might have to account for all *Ezhip* homologs in mice, or an alternate model system (*e.g.,* rats) lacking such a proliferation of paralogs might be more appropriate.

Finally, our findings may also have implications for EZHIP’s role in cancer. *Ezhip* was discovered initially due to its mutation or misexpression in cancers, especially in Posterior fossa type A (PFA) ependymomas (Dewaele et al., 2014; Hübner et al., 2019; Pajtler et al., 2018). Incidentally, similar misexpression of short histone H2A variants has also been shown in some cancers (Chew et al., 2021). Our finding of *Ezhip2*, which encodes an intact KLP motif, raises the possibility that it may also be mis-expressed in some cancers, where it might drive cancer progression even though it encodes a truncated protein. Indeed, fusions between EZHIP truncations and transcription factors have been predicted to result in aberrant PRC2 localization in cancers (Dewaele et al., 2014). One such fusion identified in endometrial stromal sarcomas occurred between motifs 8–10 of EZHIP and MBTD1, a chromatin reader of the NuA4 histone acetyltransferase complex (Dewaele et al., 2014). Our discovery of *Ezhip*2, which also encodes most of motifs 8–10, thus opens new avenues to study its role upon misexpression, either by itself or as a fusion protein, in cancer.

## Methods

### Identification of EZHIP orthologs

To identify EZHIP orthologs, we iteratively queried the genomes of 68 placental mammals (see species list in Supplementary Table S1) with representation from primates, glires, ungulates, carnivores, bats, Afrotheria, and Xenarthra. We also queried two non-placental mammalian outgroup species: the gray short-tailed opossum (*Monodelphis domestica*) and the platypus (*Ornithorhynchus anatinus*). We used TBLASTN (Altschul et al., 1990; Altschul et al., 1997) to perform a homology-based search starting with human EZHIP as our query. We used genomes from NCBI’s non-redundant nucleotide collection (nr/nt) and whole-genome shotgun contig (wgs) databases to query for homology.

In some mammals, a homology search with human EZHIP did not yield any results due to poor homology. This was particularly true of Glires. In these cases, we identified EZHIP orthologs using two complementary approaches. First, we ran syntenic analyses to identify the genomic neighborhood where EZHIP is expected to be found. Second, we used EZHIP sequences from more closely related species as TBLASTN queries to determine EZHIP orthologs, or, in the case of mouse, we used existing literature (Ragazzini et al., 2019). Using the identified orthologs, we queried the genome again to identify any paralogs. For example, using mouse EZHIP to query the mouse genome led us to identify 15 additional homologous EZHIP sequences. In all cases, paralogs were reciprocally blasted against *Homo sapiens* or other closely related species to determine if the identified paralogs were EZHIP-related. Furthermore, all orthologs and paralogs with intact open reading frames were included in our maximum-likelihood phylogenetic analyses described below to confirm their relationship to EZHIP.

We examined the shared synteny (conserved genetic neighborhood) of EZHIP to confirm orthology and to date its origin. Syntenic analyses were performed in two non-placental mammalian outgroup species (*Monodelphis domestica* and *Ornithorhynchus anatinus*) and 15 placental mammals: human (*Homo sapiens)*, mouse (*Mus musculus)*, rat (*Rattus norvegicus)*, guinea pig (*Cavia porcellus*), rabbit (*Oryctolagus cuniculus)*, pig (*Sus scrofa*), sheep (*Ovis aries)*, cow (*Bos taurus)*, horse *(Equus caballus)*, south-central black rhinoceros (*Mirounga leonina)*, cat *(Felis catus)*, dog (*Canis lupus familiaris)*, greater horseshoe bat (*Rhinolophus ferrumequinum*), Asian elephant (*Elephas maximus)*, and armadillo (*Dasypus novemcinctus*). Using the UCSC Genome Browser (Kent et al., 2002), genomic neighborhoods were identified for all our hits. EZHIP is present on the X chromosome, which is more susceptible to evolutionary turnover. Therefore, we identified well-conserved flanking genes surrounding EZHIP (BMP15, NUDT10/11, MAGED1) using the Genomicus browser (Nguyen et al., 2022). Using TBLASTN, the flanking genes were used as queries to identify syntenic regions across the 17 mammalian genomes (Figure 1B). In some cases, the syntenic location was split across multiple scaffolds, indicated by double slashes in Figure 1.

EZHIP orthologs were identified based on sequence homology to known EZHIP sequences (predominantly human or rodent), conserved synteny, and phylogenetic relationships (below). Sequences that did not meet these criteria were classified as paralogs or pseudogenes. Pseudogenes were annotated based on the presence of multiple stop codons, frameshifts that disrupt open reading frames, or the absence of any initiating methionine (Supplementary Table S1).

### Phylogenetic analyses

Phylogenetic analyses were performed to resolve the relationships among all identified EZHIP homologous sequences, distinguishing orthologs from paralogs. The MUSCLE algorithm (Edgar, 2004) in Geneious Prime 2025.0.3 (https://www.geneious.com) was used to perform protein and nucleotide alignments. Positions present in fewer than half of all sequences were removed from the alignment (de-gapped) to allow for a more stringent analysis, and pseudogenes were also excluded (Supplementary Data S1 and S5). Phylogenetic trees were constructed using maximum-likelihood methods implemented in PhyML (Guindon et al., 2010; Guindon & Gascuel, 2003) and the JTT substitution model (Jones et al., 1992) with 100 bootstrap replicates (Figure 1C, Supplementary Figure S8).

### Calculating the rate of protein divergence

Pairwise protein sequence identities were calculated for EZHIP and the primate EZH2 across representative species of primates, carnivores, ungulates, or rodents, using the human, dog, goat, or mouse orthologs as references, respectively. (Figure 3; Supplementary Table S2). Median species divergence times were obtained from the TimeTree database (www.timetree.org (Hedges et al., 2015)).

### Analysis of evolutionary selective pressures

We analyzed selective pressures on EZHIP and EZHIP2 across diverse mammals and simian primates using the codeml algorithm from the PAML suite (Yang, 1997) (Supplementary Data S3, S4, and S6). Codon alignments were generated and further manually refined where necessary. Alignments were trimmed to remove gaps or regions unique to a single species. The resulting alignment was used to construct a phylogenetic tree using PhyML with maximum-likelihood methods and the GTR substitution model (Guindon et al., 2010).

We assessed gene-wide purifying selection (Supplementary Table S3) using codeml model 0. This model assumes a single evolutionary rate across all lineages present in the sequence alignment. We compared the likelihoods between model 0 with *dN/dS* fixed at 1 (neutral evolution) and model 0 with *dN/dS* estimated from the alignment. Statistical significance was determined by comparing twice the difference in log-likelihoods between the two models to a χ^2^ distribution with one degree of freedom (Yang, 1997).

To test for site-specific positive selection (Supplementary Table S4), we compared two pairs of PAML NSsites models. We compared log likelihoods between model 8 (which encompasses 10 codon categories with *dN/dS* values between 0 and 1 and an additional category with *dN/dS* > 1) and either model 7 (which restricts *dN/dS* to values below 1) or model 8a (which fixes the additional category at *dN/dS = 1*). Statistical significance was determined by comparing twice the difference in log-likelihoods between the models to a χ^2^ distribution, with degrees of freedom corresponding to the difference in the number of model parameters (Yang, 1997). We categorize positively selected sites as those that have a Bayes Empirical Bayes (BEB) posterior probability greater than 90% in model 8. To validate our findings, we conducted an independent analysis using the FUBAR algorithm (Murrell et al., 2013) in Datamonkey (https://www.datamonkey.org), which estimates selection at individual sites and across the entire gene (Supplementary Table S4).

### Motif analyses

Ten motifs were identified using both MEME and MAST (Bailey et al., 2015) on full-length EZHIP protein sequences from more than 70 placental mammals (Supplementary Data S2). For downstream analyses with MEME, MAST, and TRF, a single representative copy from each species was selected.

While motifs 1–5 and 8–10 were typically found only once in each EZHIP homolog, motifs 6 and 7 were highly repetitive. Therefore, they were excluded from initial analyses to prevent faulty conclusions regarding retention or loss and were instead analyzed for tandem repeat characteristics (detailed below). All identified motifs had E-values < 10^−5^. In a few cases, only one program (MAST or MEME) detected a motif; these instances are shown as empty dotted boxes in Supplementary Figure S3 and with an apostrophe (‘) in Figure 2. In cases where either program did not detect motifs, all sequences within a given clade (e.g., carnivores) were aligned to manually confirm the absence of a motif. If the pairwise identity exceeded 50%, we manually classified this motif as present. In addition, the entire human EZHIP sequence was used to identify a nuclear localization sequence (NLS) using PSORT (Nakai & Horton, 1999) (https://psort.hgc.jp/). This NLS overlaps with a well-conserved stretch in Motif 5.

Motifs 6 and 7 were highly repetitive across species. Therefore, we analyzed 50 species using Tandem Repeat Finder (TRF) (https://tandem.bu.edu/trf/trf.html) (Benson, 1999), assessing repeat length, copy number, and sequence similarity. For each sequence, non-overlapping repeats were annotated based on the minimum repeat length. We further annotated repeat sequences to overlap with the open reading frame. We then used iterative alignments between human and each species or between species within each clade to annotate and classify repeats as mammal-wide (suggesting an origin in the last common ancestor of all mammals), clade-specific, or species-specific. However, given the mutation-prone nature of repeats, we cannot eliminate the possibility that species- or even clade-specific repeats originated from the ancestral repeat sequence (Figure 2). Motif logo plots (Figure 2C, D, and Supplementary Figure S4) were created from these alignments using WebLogo ((Crooks et al., 2004) weblogo.berkeley.edu).

### RNA-seq analysis

Publicly available transcriptome data from human tissues (Brawand et al., 2011; Williams et al., 2015), rhesus macaque tissues (Brawand et al., 2011), human spermatogenesis (Jan et al., 2017), and human embryo (Hendrickson et al., 2017) were analyzed to quantify the expression of *Ezhip* and *Ezhip2* (87.4% identical to EZHIP) in somatic and germline tissues (Supplementary Table S5). FASTQ files were downloaded using NCBI’s SRA toolkit (https://www.ncbi.nlm.nih.gov/books/NBK158900), and reads were mapped to species-specific genome assemblies using STAR (Dobin et al., 2013) with the options –outMultimapperOrder Random –outSAMmultNmax 1 –twopassMode Basic to randomly assign multimapping reads to a single location. Gene-level read counts were obtained using genomic coordinates of each ORF and BEDTools multicov (Quinlan & Hall, 2010). RPKM values were calculated by dividing read counts by the total number of mapped reads (in millions) and transcript length (in kilobases).

## Supplementary Material

Supplement 1

## Data Availability

Supplementary Data files include all sequences used in trees and all mammalian orthologs and paralogs of EZHIP described in the paper.
