# Supplementary material for: Dynamic evolution of EZHIP, an inhibitor of the Polycomb Repressive Complex 2 in mammals
Source: bioRxiv. 2025 Dec 12:2025.12.12.693809. Preprint. [Version 1] doi: 10.64898/2025.12.12.693809 (PMC12713659; doi:10.64898/2025.12.12.693809)
Supplement: Supplement 1 [file media-1.zip › Supplementary materials/EZHIPEvolution_BiorxivSupplementaryFigures.pdf]

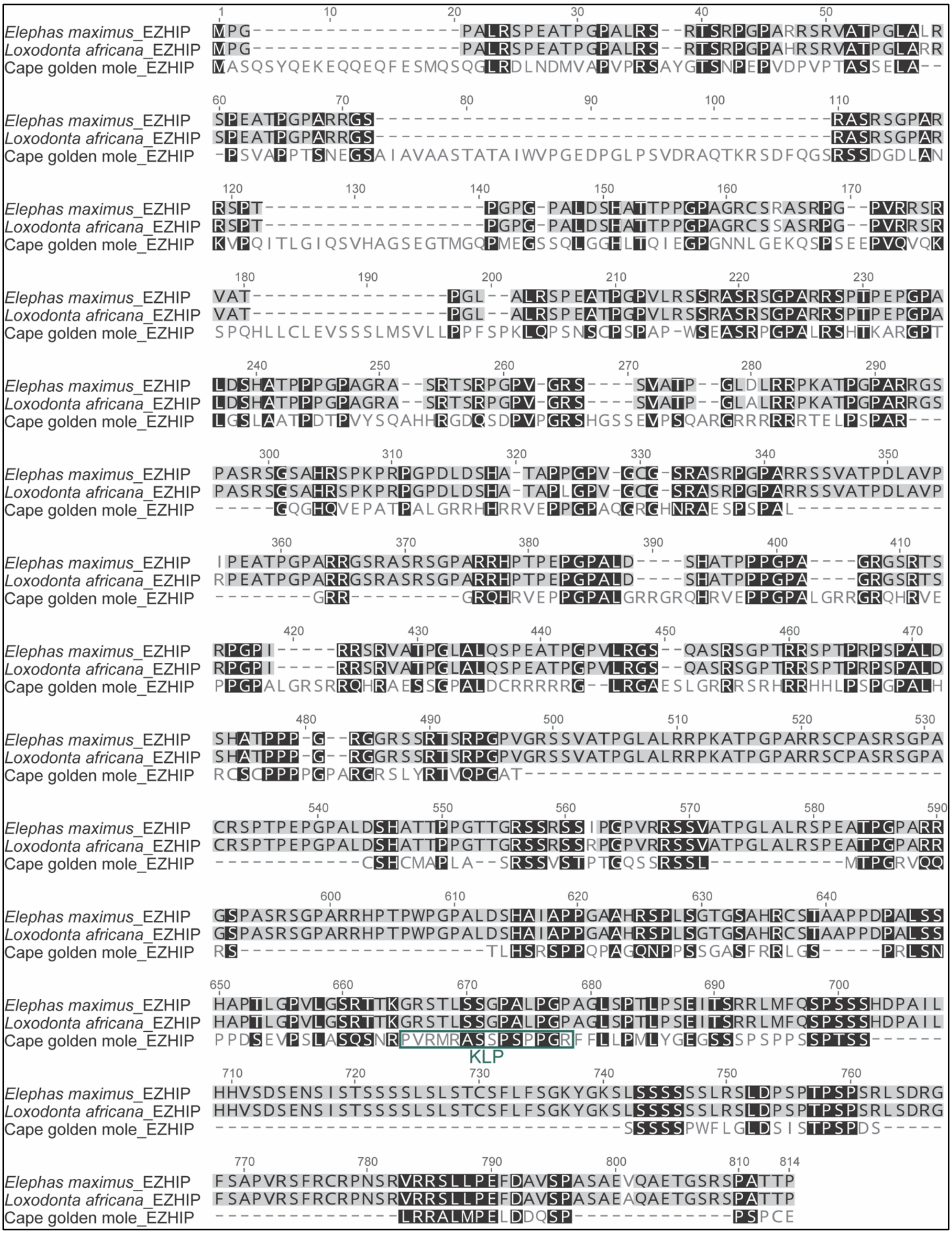

Supplementary Figure S1. Protein sequence alignment of Afrotherian species

A. EZHIP from two elephant species, *Loxodonta africana* and *Elephas maximus*, and from the Cape golden mole is shown. Residues with black and grey backgrounds indicate high sequence similarity. The KLP motif in the Cape golden mole is indicated with a dark green outlined box.

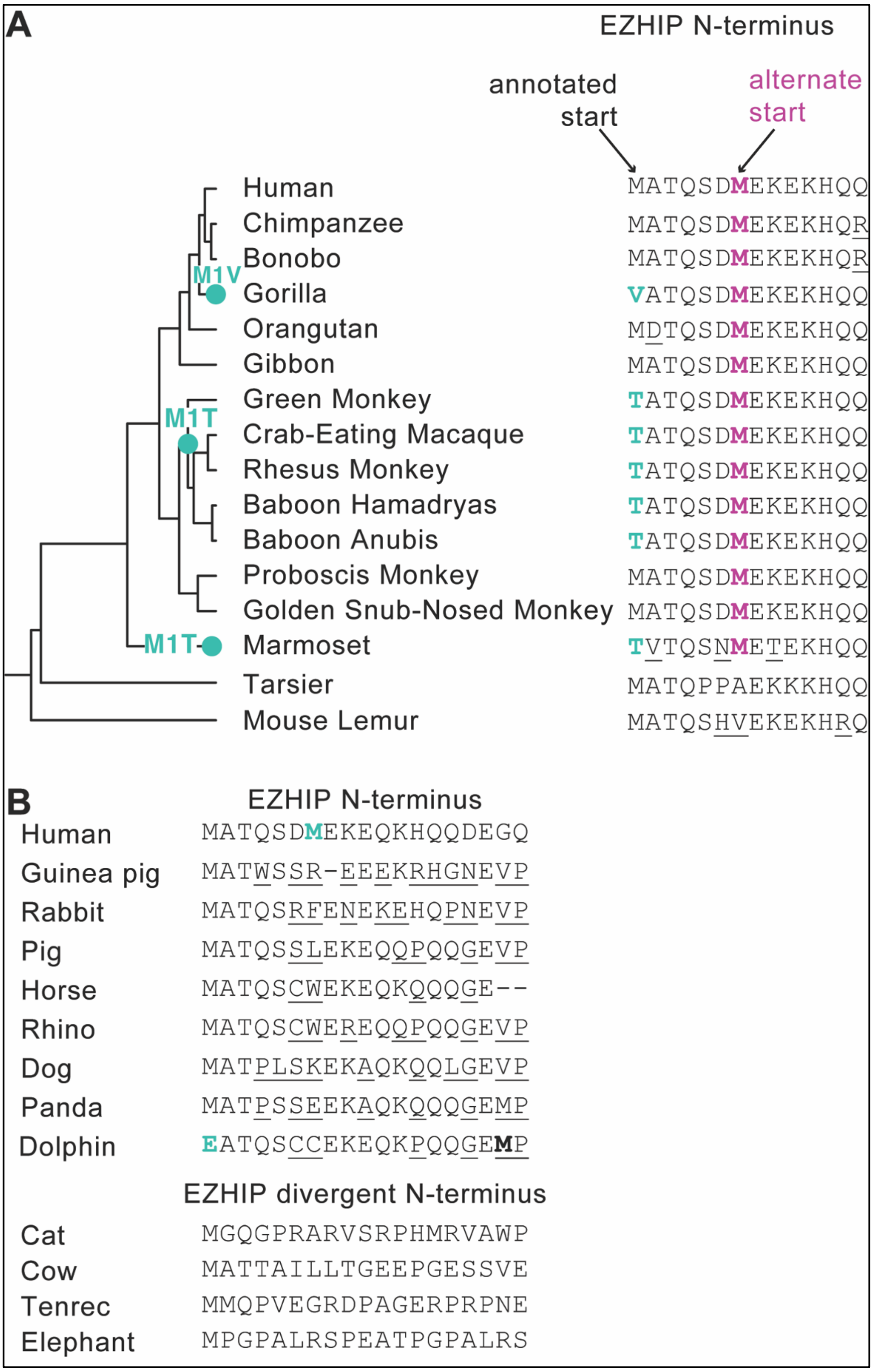

Supplementary Figure S2. Multiple primates and several additional mammals may have evolved an alternate start codon A. The first 14 amino acids of primate EZHIP are shown beside a primate species tree. Repeated loss of the previously annotated start codon is indicated with a teal dot and the mutation. A conserved, potentially alternate start is

indicated in pink within the sequence. **B.** The first 17 amino acids of EZHIP in representative mammals are shown. Dolphin also has an alternate start (boldface), and cat, cow, tenrec, and elephant have a divergent N-terminal tail.

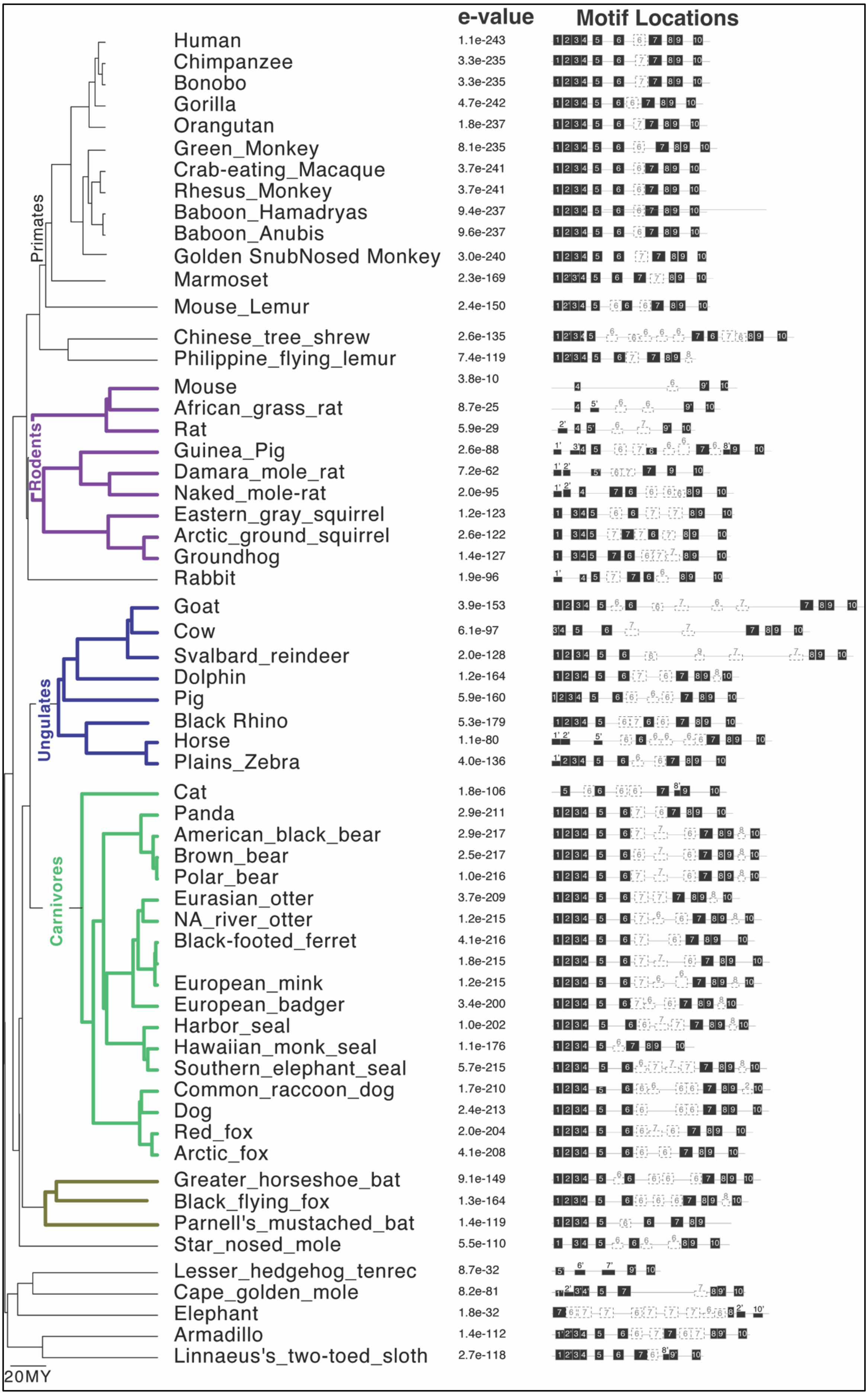
e-value
Motif Locations
Primates
Rodents
Ungulates
Carnivores
20MY
Human 1.1e-243
Chimpanzee 3.3e-235
Bonobo 3.3e-235
Gorilla 4.7e-242
Orangutan 1.8e-237
Green_Monkey 8.1e-235
Crab-eating_Macaque 3.7e-241
Rhesus_Monkey 3.7e-241
Baboon_Hamadryas 9.4e-237
Baboon_Anubis 9.6e-237
Golden SnubNosed Monkey 3.0e-240
Marmoset 2.3e-169
Mouse_Lemur 2.4e-150
Chinese_tree_shrew 2.6e-135
Philippine_flying_lemur 7.4e-119
Mouse 3.8e-10
African_grass_rat 8.7e-25
Rat 5.9e-29
Guinea_Pig 2.6e-88
Damara_mole_rat 7.2e-62
Naked_mole-rat 2.0e-95
Eastern_gray_squirrel 1.2e-123
Arctic_ground_squirrel 2.6e-122
Groundhog 1.4e-127
Rabbit 1.9e-96
Goat 3.9e-153
Cow 6.1e-97
Svalbard_reindeer 2.0e-128
Dolphin 1.2e-164
Pig 5.9e-160
Black Rhino 5.3e-179
Horse 1.1e-80
Plains_Zebra 4.0e-136
Cat 1.8e-106
Panda 2.9e-211
American_black_bear 2.9e-217
Brown_bear 2.5e-217
Polar_bear 1.0e-216
Eurasian_otter 3.7e-209
NA_river_otter 1.2e-215
Black-footed_ferret 4.1e-216
1.8e-215
European_mink 1.2e-215
European_badger 3.4e-200
Harbor_seal 1.0e-202
Hawaiian_monk_seal 1.1e-176
Southern_elephant_seal 5.7e-215
Common_raccoon_dog 1.7e-210
Dog 2.4e-213
Red_fox 2.0e-204
Arctic_fox 4.1e-208
Greater_horseshoe_bat 9.1e-149
Black_flying_fox 1.3e-164
Parnell's_mustached_bat 1.4e-119
Star_nosed_mole 5.5e-110
Lesser_hedgehog_tenrec 8.7e-32
Cape_golden_mole 8.2e-81
Elephant 1.8e-32
Armadillo 1.4e-112
Linnaeus's_two-toed_sloth 2.7e-118

Supplementary Figure S3. EZHIP has eight conserved motifs and tandem repeats across placental mammals. A. A mammalian species tree (colored as in Fig. 1C) is shown alongside a representative protein schematic with identified motifs that are typically found as one copy (black filled boxes with numbers 1-5 and 8-10) or in multiple copies (motifs 6 and 7). Motif heights represent the significance of a motif site within the sequence, with taller motifs representing more statistically significant sites. Motifs that were repeated more than once in an EZHIP, most notably motif 6 and 7, are represented as a box with dashed lines. Motifs only identified by one program (MAST or MEME) are indicated with an apostrophe. E-values are indicated to the left of motif schematics. See Methods for details.

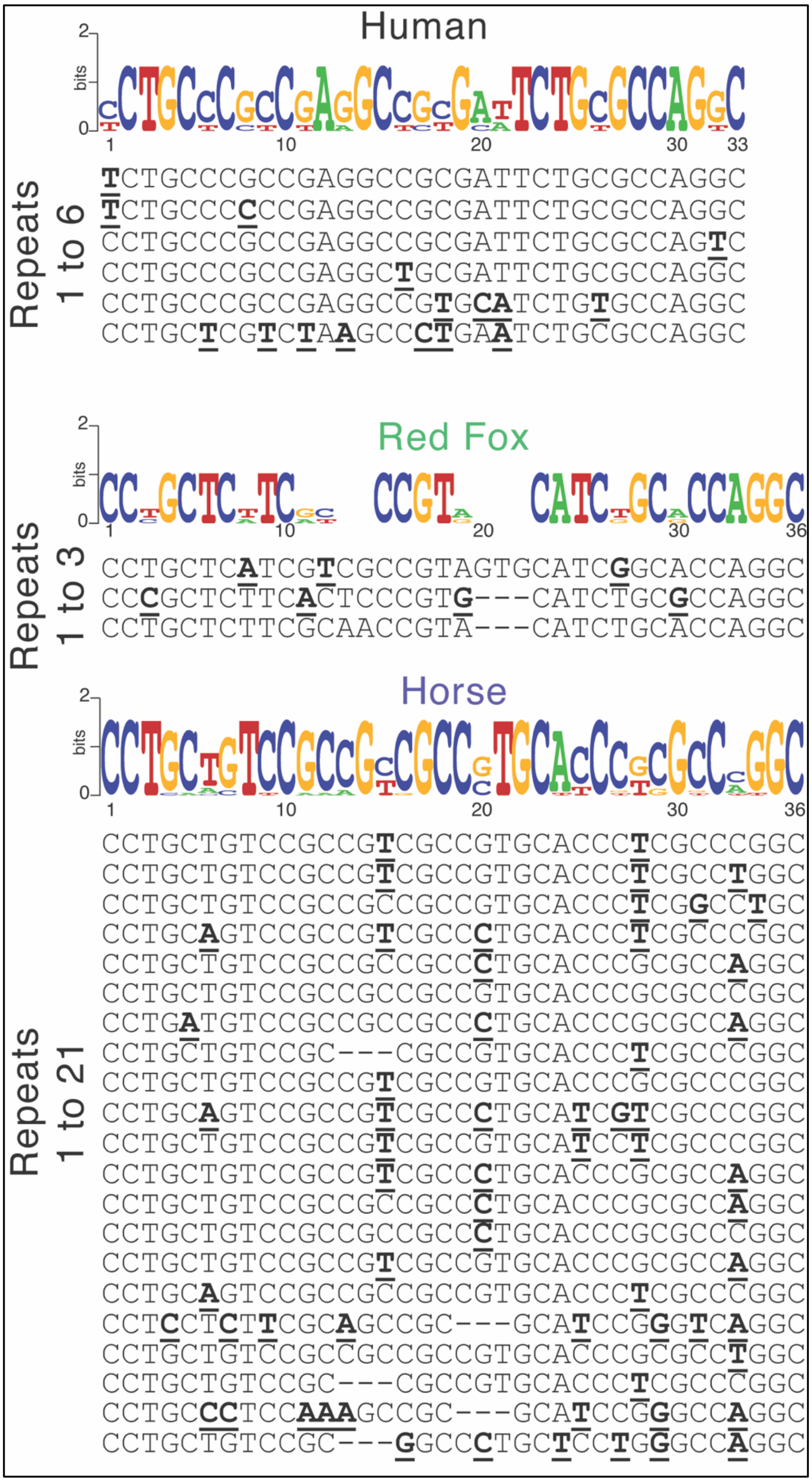

Supplementary Figure S4. Repeats are conserved within mammalian species.

Alignment of the tandem repeats in human, red fox, and horse. Logos plots as in Fig. 2.

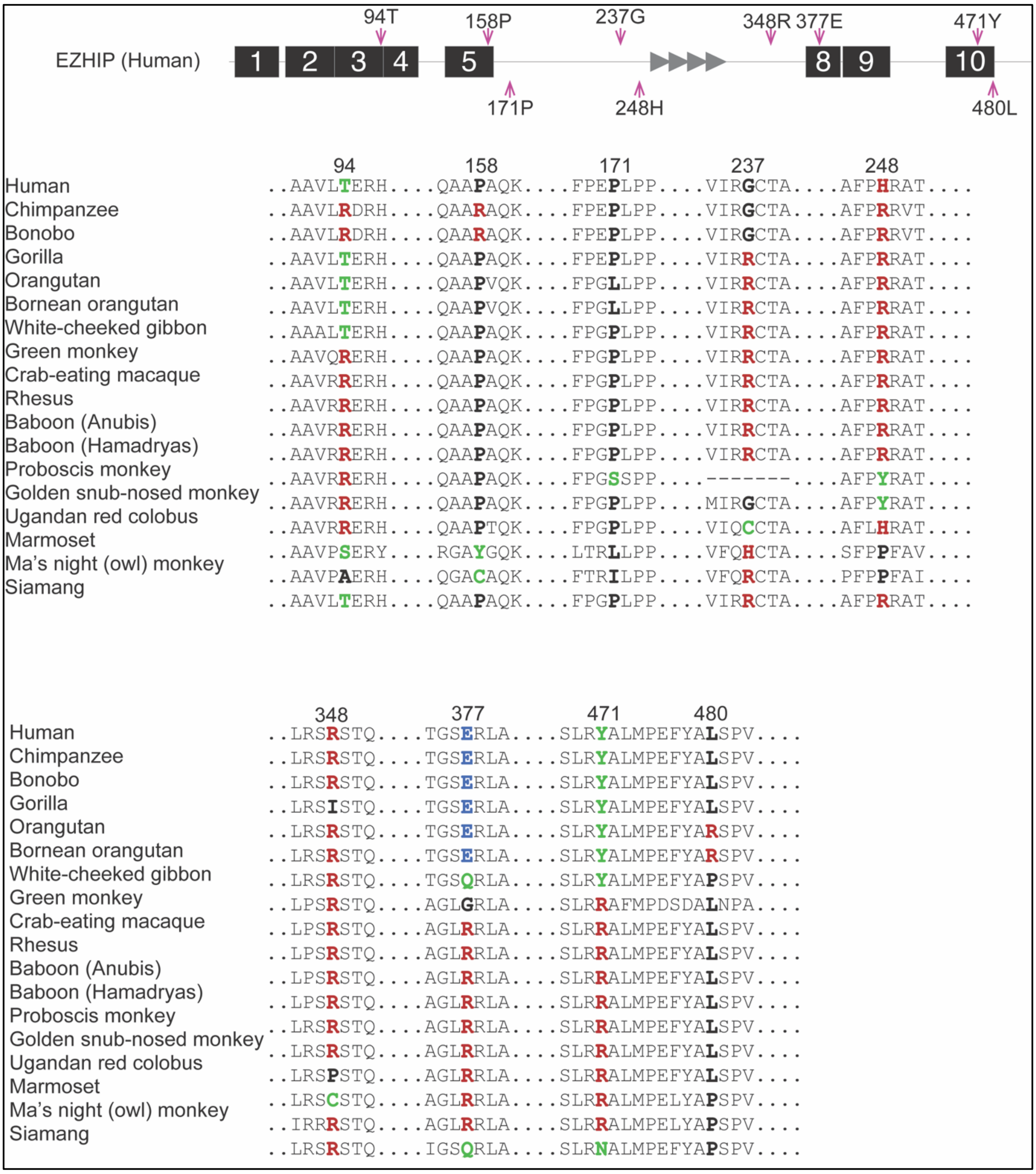

Supplementary Figure S5. Evidence for positive selection at a subset of sites across simian primate EZHIP. Alignments of regions surrounding positively selected sites (colored amino residues) in EZHIP across simian primates identified by PAML. Colors of residues highlight their biochemical properties: hydrophobic (black), negatively charged (blue), positively charged (red), and polar (green). Also see Figure 3B.

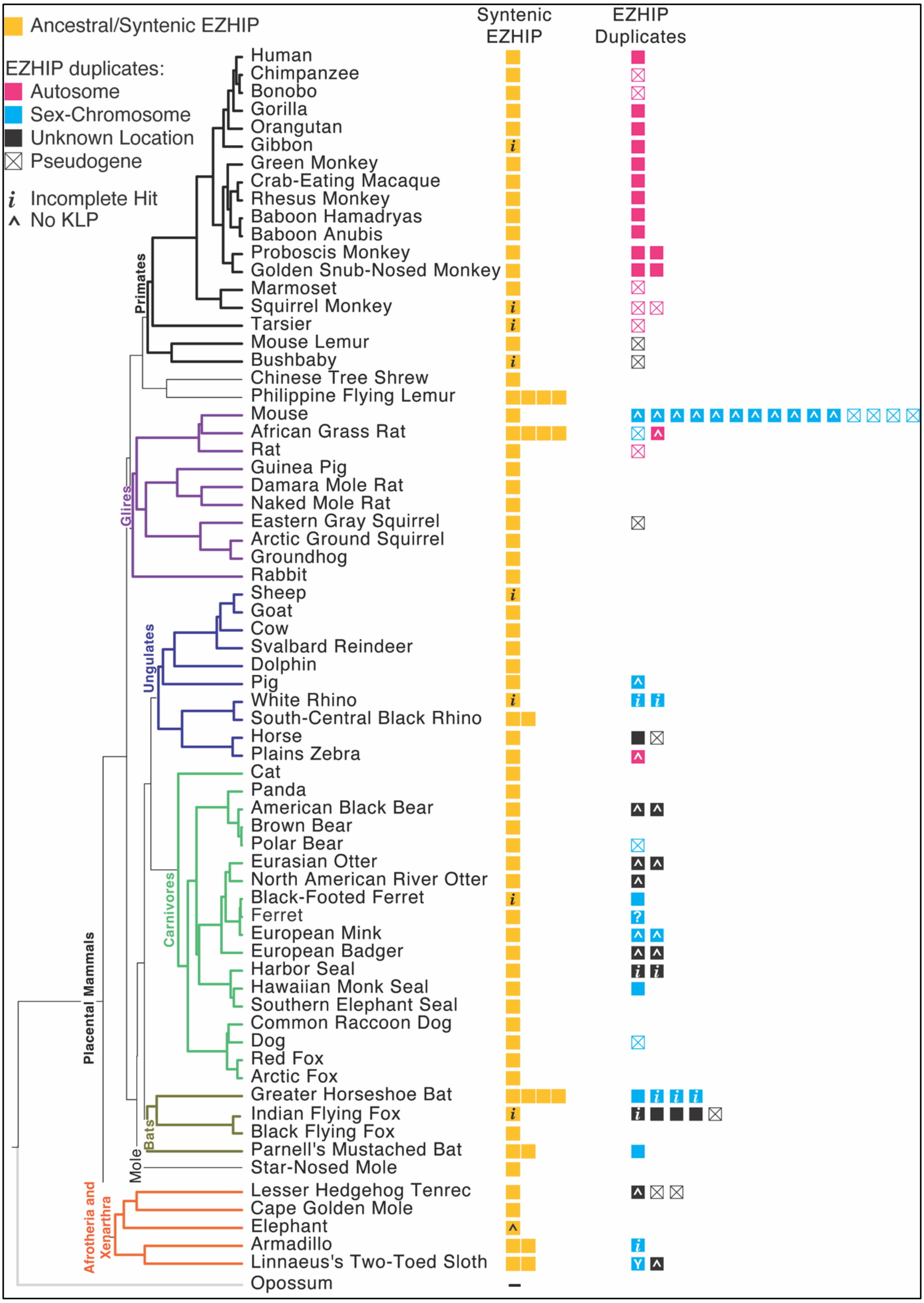

Supplementary Figure S6. EZHIP has repeatedly duplicated in mammals. EZHIP copies are represented as colored boxes alongside a mammalian species tree (colored as in Fig. 1C). Orthologs and duplicates in the same syntenic location are shown as yellow-filled

boxes. EZHIP duplicates can be found on autosomes (pink boxes), sex chromosomes (blue boxes), or at unknown chromosomal locations (black boxes). Most sex chromosome duplicates are found on the X chromosome, except for the duplicate in Linnaeus's two-toed sloth that is found on the Y chromosome. Boxes containing an X represent putative pseudogenes, i represents incomplete sequences due to gaps in genome assembly, and ^ represents a lack of a KLP sequence. In one case, a ferret duplicate, we are unable to confirm if the sequence has coding potential due to variability in the population; therefore, it is represented with a ?.

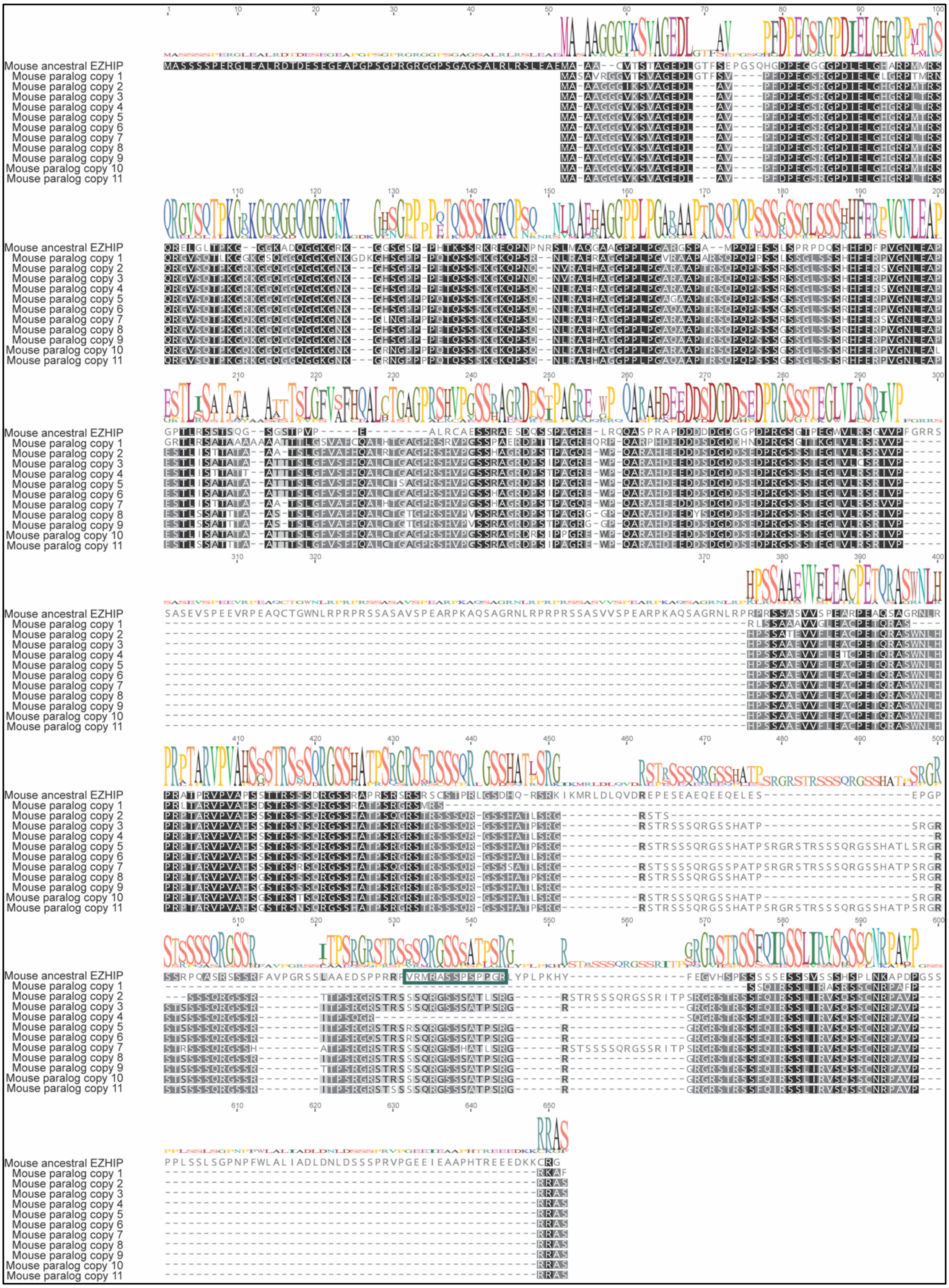

Supplementary Figure S7. Mouse carries 11 EZHIP paralogs that lack the KLP. The mouse EZHIP ortholog is compared to 11 EZHIP paralogs identified in the mouse genome. Residues with black and grey backgrounds indicate high sequence similarity, and a LOGO plot showing sequence conservation is displayed above the alignments. The KLP motif,

found only in the ancestral mouse EZHIP (between residues 530 and 550 in the alignment), is indicated with a green box.

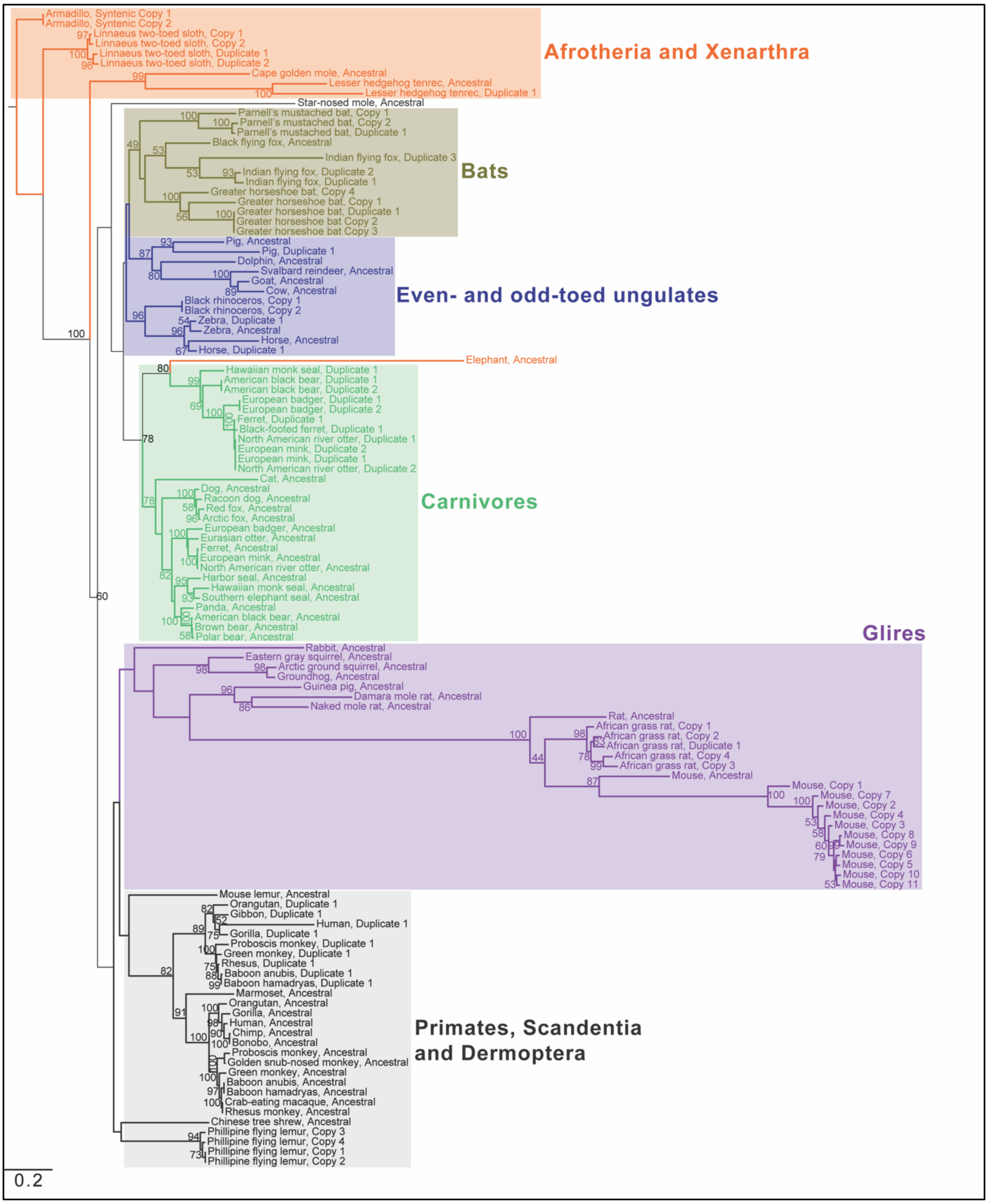

Supplementary Figure S8. Phylogeny of mammalian EZHIP orthologs and paralogs. Maximum-likelihood protein phylogenetic tree of EZHIP from 70 representative mammalian species (colored as in Figure 1C, Supplementary Data S5). Bootstrap values at selected nodes with >50% support are shown. Names of mammals are indicated at branch tips. In species with multiple copies of EZHIP, ancestral copies identified based on syntenic location are indicated with ‘Ancestral’, syntenic copies that could not be reliably distinguished from ancestral copies are indicated with ‘Copy #’, and duplicate copies that could be reliably

distinguished from ancestral EZHIP are indicated with ‘Duplicate #’. The bottom-left scale bar below the phylogeny represents a time of 0.2 substitutions per site.

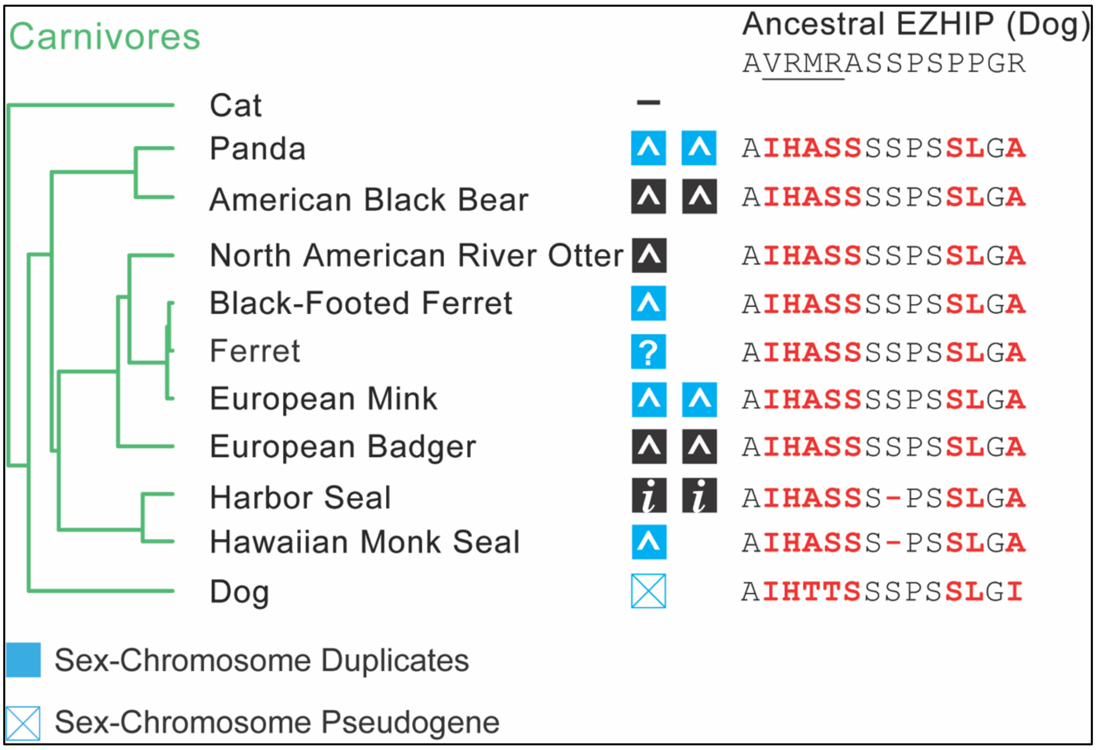

Supplementary Figure S9. An EZHIP paralog arose in the last common ancestor of carnivores. Left, an EZHIP paralog that can be found in the same syntenic location in carnivores and that has been duplicated in carnivores is illustrated with a box alongside a carnivore species tree. While these duplicates are syntenic, blue boxes indicate identification on the X chromosome, while black boxes indicate inability to pinpoint exact chromosomal location due to poor genome assembly. Boxes containing an X represent putative pseudogenes, i represents incomplete sequences due to gaps in genome assembly, and ^ represents a lack of a KLP sequence in duplicates. Duplicate(s) were not identified in cat through homology or synteny analyses. Right, the KLP sequence with the oncohistone-mimic region underlined from dog ancestral EZHIP (top) is compared to the same region across the paralog, with changes relative to ancestral EZHIP in red.

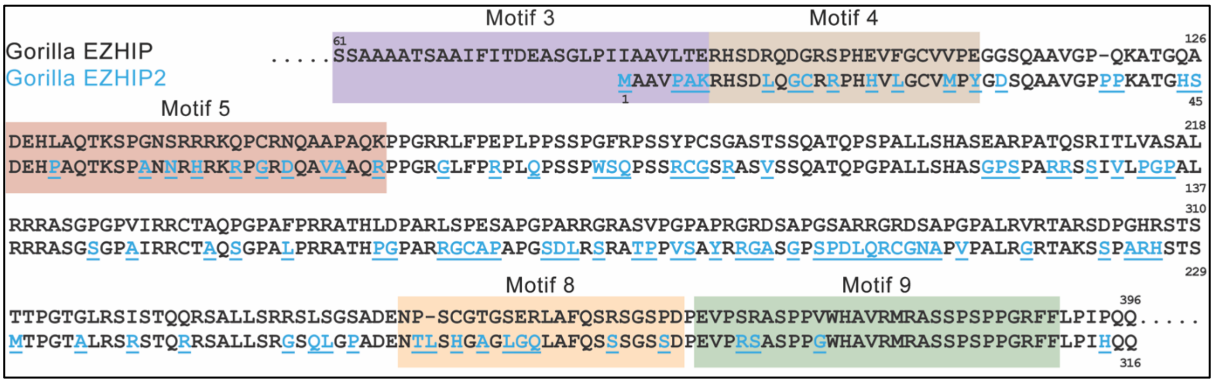

Supplementary Figure S10. EZHIP and EZHIP2 show ~65% identity. An alignment of Gorilla EZHIP and EZHIP2 is shown with motifs 3-5 and motifs 8 and 9 highlighted in various colors. Residues that are different between EZHIP and EZHIP2 are colored in blue.

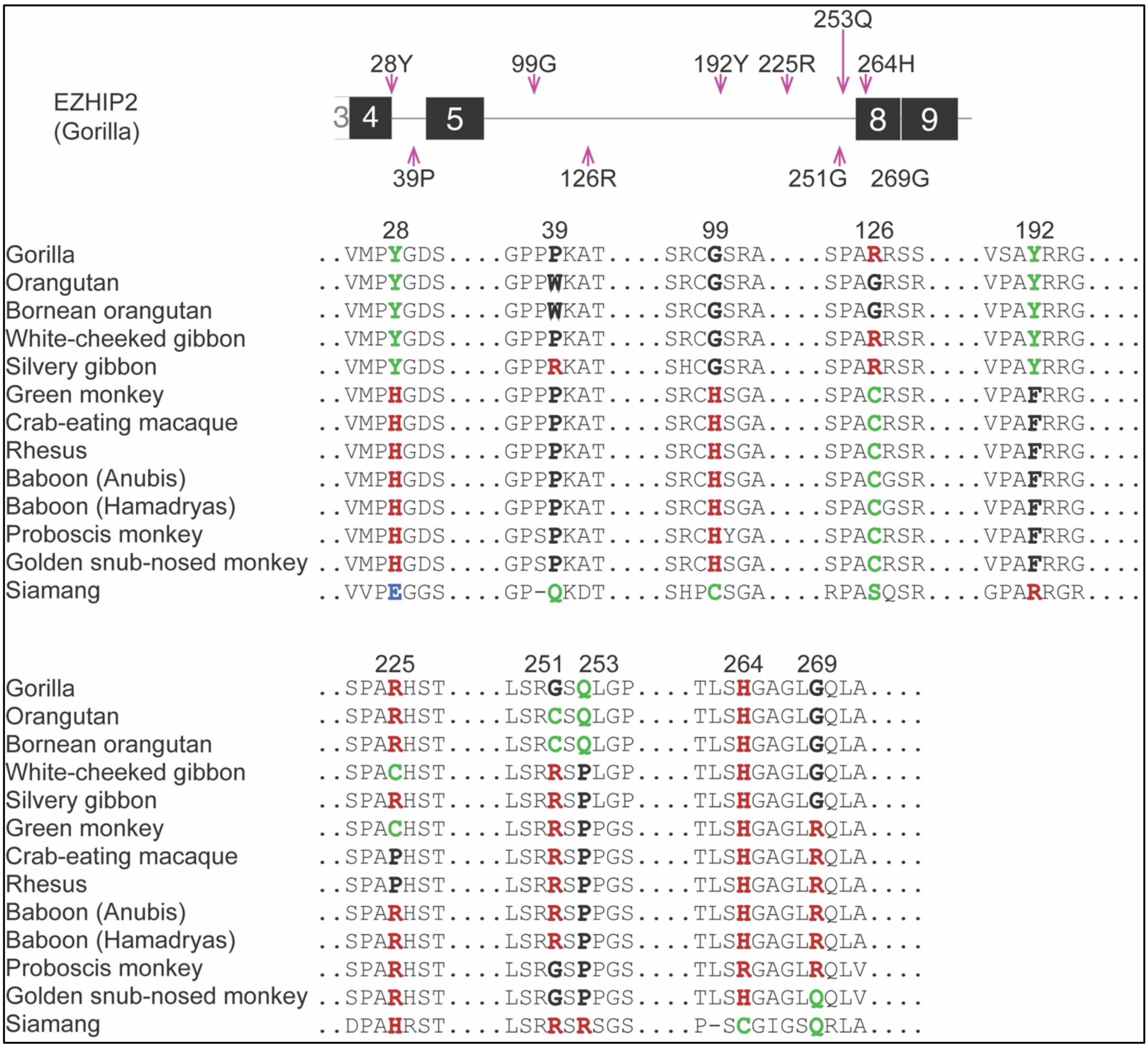

Supplementary Figure S11. Evidence of positive selection at a subset of sites across simian primate EZHIP2. Alignments of regions surrounding positively selected sites (colored amino residues) in simian primate EZHIP2 identified by PAML. Colors of residues highlight their biochemical properties: hydrophobic (black), negatively charged (blue), positively charged (red), and polar (green). Also see Figure 4.
